# A global systematic review of frugivorous animal tracking studies and the estimation of seed dispersal distances

**DOI:** 10.1002/ece3.10638

**Published:** 2023-10-31

**Authors:** Adam Fell, Thiago Silva, A. Bradley Duthie, Daisy Dent

**Affiliations:** ^1^ Biological and Environmental Sciences University of Stirling Stirling UK; ^2^ Department of Environmental Systems Science Institute of Integrative Biology, ETH Zurich Zurich Switzerland; ^3^ Max Planck Institute for Animal Behaviour Konstanz Germany; ^4^ Smithsonian Tropical Research Institute Balboa Panama

**Keywords:** animal behaviour, animal movement, frugivore, GPS transmitter, radio transmitter, seed dispersal, tracking

## Abstract

Seed dispersal is one of the most important ecosystem functions globally. It shapes plant populations, enhances forest succession, and has multiple, indirect benefits for humans, yet it is one of the most threatened processes in plant regeneration, worldwide. Seed dispersal distances are determined by the diets, seed retention times and movements of frugivorous animals. Hence, understanding how we can most effectively describe frugivore movement and behaviour with rapidly developing animal tracking technology is key to quantifying seed dispersal. To assess the current use of animal tracking in frugivory studies and to provide a baseline for future studies, we provide a comprehensive review and synthesis on the existing primary literature of global tracking studies that monitor movement of frugivorous animals. Specifically, we identify studies that estimate dispersal distances and how they vary with body mass and environmental traits. We show that over the last two decades there has been a large increase in frugivore tracking studies that determine seed dispersal distances. However, some taxa (e.g. reptiles) and geographic locations (e.g. Africa and Central Asia) are poorly studied. Furthermore, we found that certain morphological and environmental traits can be used to predict seed dispersal distances. We demonstrate that flight ability and increased body mass both significantly increase estimated seed dispersal mean and maximum distances. Our results also suggest that protected areas have a positive effect on mean seed dispersal distances when compared to unprotected areas. We anticipate that this review will act as a reference for future frugivore tracking studies, specifically to target current taxonomic and geographic data gaps, and to further explore how seed dispersal relates to key frugivore and fruit traits.

## Graphical abstract

We demonstrate that an increase in body mass significantly increases the estimated seed dispersal mean and maximum distances, as does species flight ability. Our results also suggest that protected areas have a positive effect on mean seed dispersal distances when compared to unprotected areas. We anticipate that this review will act as a reference for future frugivore tracking studies, specifically to target current taxonomic and geographic data gaps, and to further explore how seed dispersal relates to key frugivore and fruit traits.

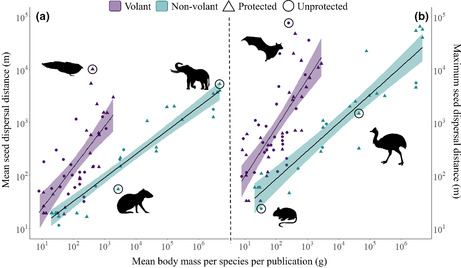

## 1 INTRODUCTION

Seed dispersal is one of the most important ecosystem functions globally (Aslan et al., 2013). Seed dispersal plays a pivotal role in shaping plant populations by facilitating regeneration through the movement of seeds and subsequent plant recruitment. Additionally, gene flow is influenced by the movement of alleles driven by seed dispersal, further contributing to local population dynamics and genetic diversity. (Jansen et al., 2008; Jordano et al., 2011). Humans indirectly benefit from this global service through the seed dispersal of valuable timber species, and edible and medicinal plants (Wenny et al., 2016), yet seed dispersal is one of the most threatened processes in plant regeneration, worldwide (Neuschulz et al., 2016). Habitat loss and fragmentation are the main threats to seed dispersal as they restrict the movement and natural behaviour of local seed dispersers (Browne & Karubian, 2018; Mahoney et al., 2018). Accurate measurement of seed dispersal distances is essential to fully understand the effect of habitat loss on critical ecosystem functions.

Over half of woody plant species globally, and up to 90% of tropical tree species, require animals to disperse their seeds (Howe & Smallwood, 1982). Animal‐mediated seed (or diaspore) dispersal can take many forms, including endozoochory (carried within an animal), epizoochory (attached to the outside of a disperser), and synzoochory (intentionally carried, mostly in the mouth). The way seeds are transported can often help predict the fate of the seed (Nascimento et al., 2020), but the decisions that animals make relating to movements before, during and after interacting with fruit ultimately drive the dynamics of animal dispersed plant populations (Morales et al., 2013). These decisions are shaped by landscape composition, animal traits, diet preferences and behaviours (Baguette & Van Dyck, 2007). Even decisions that are not directly related to foraging, for example, use of leks, latrines or roosting, can incidentally impact the deposition of seeds through altered movement paths (Sasal & Morales, 2013).

Recent studies call for animal movement and behaviour to be better integrated with seed dispersal studies to enable researchers to fully understand the processes that determine seed rain (Borah & Beckman, 2022; Côrtes & Uriarte, 2013) and to advance a mechanistic understanding of animal‐mediated seed dispersal. For example, interdisciplinary collaborations linking plant demography and movement ecology could use animal tracking studies to determine the precise location of seed deposition and to describe the dispersal potential of different frugivorous animal species (Borah & Beckman, 2022; Dent & Estrada‐Villegas, 2021).

Since the early 1990s, researchers have used tracking technology to study frugivore movement. Animal movement studies have increased exponentially in the last two decades due to the continued advancement of animal tracking and biologging technology (Kays et al., 2015; Nathan et al., 2022; Williams et al., 2020). Recent GPS miniaturisation has enabled tracking studies to focus on smaller animals, while previous tracking was constrained to larger species to meet tag size requirements (Wild et al., 2022). In addition, the development of solar powered tags and remote downloading has enabled long‐term studies and allowed researchers to track more species in more remote habitats (Bridge et al., 2011; Flack et al., 2016). Such developments make understanding seed dispersal through the lens of movement ecology more accessible and plausible, and increasingly, studies have used tracking data to infer seed dispersal effectiveness (Hirsch et al., 2012; Holbrook & Smith, 2000; Kays et al., 2011; Rehm et al., 2019). Most commonly, studies infer the movement of seeds using distances travelled during seed retention time (the time the seed is retained by a frugivore, that is, often the time taken for seeds to move through the gut). Simulated GPS tracks are predicted for the species‐specific seed retention time using the fitted distributions of actual animal movement, which can then be used to fit seed dispersal kernels (Nathan & Muller‐Landau, 2000).

Seed dispersal is defined by (1) frugivore diet, (2) seed retention time and (3) movement behaviour (Morales et al., 2013; Morales & Morán López, 2022). Frugivore diets can be described by targeted observations or faecal analysis. Observational studies identify frugivore‐plant interactions directly and are a low‐cost method, but they can be subject to observer errors and bias, and require significant field effort (Matthews et al., 2020). Analysis of faecal samples can be a more efficient and accurate method for describing diet. Novel DNA metabarcoding techniques recover a short sequence of DNA that is characterised as a unique species identifier (Kress et al., 2015). This method can be used to identify plant species present in frugivore faeces and functions with minimal fragmented plant material, which is typical of faecal matter due to degradation through digestion (González‐Varo et al., 2014). This method requires a dedicated DNA barcoding sequence dataset of local plants for reference, so that the sequences can be matched, which can be prohibitive especially in highly diverse systems (Galimberti et al., 2016). Nonetheless, metabarcoding provides a highly effective new method for describing frugivore‐plant interactions for multiple species.

Describing seed retention time is complex and involves detailed observation and identification of ingestion and deposition events. This is challenging and typically requires knowledge of the foraging behaviour of the species, which often comes from hours of observational studies (Plein et al., 2013; Schleuning et al., 2011; Sorensen, 1981). Traditionally, seed retention time has been measured by direct or video observations of feeding and deposition events, either in the wild or in captive trials. However, recent advances in tracking technology have enabled development of small tags that can be ingested by larger frugivores (Beirne et al., 2019), and high‐resolution tracking tags that can identify certain behaviours through small changes in body position and movements (Wild et al., 2022). For example, accelerometers can measure small yet significant changes in an animal's posture to determine specific movements (Shepard et al., 2008). By pairing these with detailed observation, patterns in the acceleration data can be matched with specific behaviours, such as consumption or defecation events (Fehlmann et al., 2017).

Frugivores often have complex movement patterns but, when broken down into trajectories and integrated with seed retention times, these offer a basis for predicting the likely deposition sites of seeds (Morales et al., 2013). Frugivore movement can be measured using structured observations (Morales et al., 2013; Ramos et al., 2020), or by tracking animals with GPS or radio tracking devices (Abedi‐Lartey et al., 2016; Kays et al., 2011; Martín‐Vélez et al., 2022; Rehm et al., 2019). Structured observations refer to a systematic and organised approach to studying and recording animal movement patterns in a controlled and consistent manner, usually by selecting vantage points and positions to observe animals and by making detailed drawings of movement patterns on cells of printed maps (e.g. Ramos et al., 2020). Movement paths from tracking devices describe in more detail where an animal has travelled and, for frugivorous animals, these can be used to predict where seeds are deposited. These paths are constructed using movement models such as random walks, correlated/biased random walks and Levy walks, which use the probability distributions of movement lengths and turning angles (Michelot & Blackwell, 2019; Reynolds, 2010). Once a movement path is generated, seed shadows can be produced to determine the probability of deposition at specific distances. Seed shadows are made up of (1) Distance of seed from source, (2) Distribution and density of dispersed seeds, (3) Number of overlapping, conspecific seed shadows (Côrtes & Uriarte, 2013). Many seed shadow models use a single lognormal distribution to calculate dispersal kernels, which may not be sufficient to correctly identify spatially aggregated seed deposition patterns that are common for vertebrate seed dispersers (Russo et al., 2006). However, these models are improved by considering an animal's behavioural response to different environmental stimuli and their ability to handle potential biases within the movement data, such as spatial and temporal autocorrelation (Morales & Morán López, 2022).

The movement patterns of frugivorous animals are determined by species traits, landscape context and fruit resources. Species morphological traits define a species' functional role within an ecosystem and can impact the provisioning of ecological services. For example, large‐bodied avian frugivores are recognised as important dispersers due to the large number of seeds they disperse and their ability to disperse a diverse range of seed sizes, including large seeded species (Galetti et al., 2013; Naniwadekar, Chaplod, et al., 2019; Naniwadekar, Rathore, et al., 2019; Wotton & Kelly, 2012). Bird species gape width determines diet breadth, and species with larger gape widths tend to have a more heterogeneous diet and interact with more fruiting plants (Kitamura, 2011; Naniwadekar, Chaplod, et al., 2019; Naniwadekar, Rathore, et al., 2019; Wheelwright, 1985). Flying species are also key seed dispersers as they typically disperse seeds over longer distances and can functionally connect habitat patches in fragmented landscapes and exploit resources unavailable to terrestrial vertebrates (Borah & Beckman, 2022; Lundberg & Moberg, 2003; Şekercioğlu, 2006). The relative importance of different frugivore guilds in seed dispersal networks varies with biogeographic region and habitat (Dent & Estrada‐Villegas, 2021; García‐Rodríguez et al., 2022; Tsunamoto et al., 2020). Birds tend to be generalist and opportunistic feeders, while mammals, especially larger bodied species, can have more specialised roles and are highly important for the dispersal of larger seeds (Ong et al., 2022). Understanding how morphological traits of frugivores are linked to seed dispersal potential is a critical step in understanding the link between animal and plant communities and can help to disentangle how changes in landscape structure affect colonisation, persistence, and recovery of animal and plant communities.

An interdisciplinary approach that integrates animal movement and plant ecology is needed to better understand animal‐mediated seed dispersal, (Borah & Beckman, 2022; Dent & Estrada‐Villegas, 2021). Here we provide a comprehensive review and synthesis of the existing primary literature of global tracking studies that monitor movement of frugivorous animals, assess the current use of animal tracking in frugivory studies and provide a baseline for future studies. We identify studies that explicitly estimate dispersal distances (using estimations of habitat range, gut passage times and foraging behaviour) and assess how dispersal distances vary with animal body mass and the following environmental variables, biome, human footprint index and the presence of a protected area.

Specifically, we provide a review of all published literature that presents frugivorous animal tracking data and summarise species, location and methods used across studies. We then use the global review to assess: (i) If certain regions or taxa are over‐ or under‐represented in terms of frugivore tracking studies, (ii) How the methods used to track frugivorous animals have changed over time (iii) How environmental variables and animal species traits (here, body mass) shape seed dispersal distances among distinct frugivore taxa.

## 2 METHODS

### 2.1 Dataset collation and screening process

A literature search was conducted using Web of Knowledge and Google Scholar search engines between May and July 2020 using the key words: (animal_move* OR gps_track* OR gps_tag* OR gps_loc* OR radio_trans* OR radio_tele* OR radio_track* OR radio_tag) AND (seed_dispers* OR frugiv*). This search string generated 240 studies, of which 34 were omitted as they were data files from Figshare or Movebank data repositories (https://www.datarepository.movebank.org/), not published studies. The remaining literature was then screened for the following criteria: (1) full‐text and peer‐reviewed article in English or suitable for online translation, (2) Article presents data from radio transmitters or GPS tags attached to a predominately frugivorous animal or directly to a food resource (i.e. seed or fruit). One publication, Tamura and Hayashi (2008) could not be included as we were unable to translate the uploaded, scanned document through an online translator. We defined a frugivore following Terborgh (1986) and Fleming et al. (1987) as an animal whose diet consists of at least 50% fleshy fruits (Wilman et al., 2014). This included some largely herbivorous and omnivorous species where seeds and fruits comprised over 50% of their diet during particular seasons or life stages (i.e. pregnancy, migration, etc. Bairlein, 2002; Bodmer & Ward, 2006; Carnicer et al., 2009).

If more than one study used the same dataset, the earliest study was selected for inclusion in the review. Of the 206 studies screened, 109 met the criteria to be retained. Twelve of these studies used transmitters attached to food resources rather than frugivores directly and were retained as the information could be used to calculate seed dispersal distances. Additional articles were found in the literature cited by these articles, which had not been obtained through the previous search. This resulted in a further 39 studies that met the scientific criteria. Finally, we found a further 14 studies during a search in April 2022 using the same previous search string to incorporate studies published between the first search and the completion of the review. All studies were then screened to identify only the studies that calculated seed dispersal distances from monitoring frugivore movements through biologging techniques. A total of 162 peer‐reviewed research studies were included in the review, and 67 of these were identified as studies that estimated seed dispersal distances. No date restrictions were applied to the search, thus the earliest study included in the review was published in 1978 and the most recent in 2022. Thus, two sets of studies were used to address the study aims: (1) All 162 studies were used to investigate how frugivore tracking studies are distributed globally and how tracking methods have changed over time (aims i and ii); and (2) A subset of 67 studies were used to investigate seed dispersal distances (aim iii).

### 2.2 Data extraction

For each study, data were extracted that detailed: (1) year of study, (2) tracking method, (3) overall purpose of study, (4) country of study, (5) habitat type, (6) taxonomic group and (7) species tracked (see Appendix S1). Quantitative information including the number of individuals tracked, the average number of tracking days and the average number of location points collected per individual tag were also collated (see Appendix S2). This information was calculated from tracking summary results only when the total number of location points and deployment days were stated. Some articles did not provide sufficient information for these metrics to be calculated, and these studies were omitted from further analyses. Tracking method included either radio transmitters (VHF), GPS or resource tracking with attached radio/GPS tags. Frugivore species data included taxonomic group at a species level, the number of species studied and the number of individuals per species per study.

The mean number of tag days was calculated for each study where data were provided. This was the mean number of days reported per study where data were collected across all tracked individuals per species. The mean number of tracking locations per species per study was also calculated when this information was provided (see Appendix S2).

Mean and maximum seed dispersal distances were stated in 45 and 56 publications, respectively (see Appendix S3). When publications presented estimates for different sized seeds, different seed species or different seasons, we took an average across the different estimates as there was too much variability among studies to sub‐divide data into different seed sizes, season, or different sexes etc.

To explore what factors influenced dispersal distance, we extracted frugivore body mass and environmental variables from each study to be used as predictors for our models. Estimates of mean species body mass (g) for birds and mammals were extracted from Wilman et al. (2014). To investigate the allometric relationship between body mass and seed retention time (SRT) of the animal species within our studies, we additionally extracted seed type, seed size and SRT (minutes) from the studies that reported the required information. SRT information was extracted from a total of 42 studies and included 59 unique animal species. This information was reported differently for all studies and the SRT values were averaged across multiple seed types and sizes for an individual animal species in an individual study (Figure S1). We categorised species as either *volant* (i.e. capable of flying) or *nonvolant*. This is functionally informative, because in our data set a large proportion of the mammals studied were bat species, while birds included some flightless species such as cassowaries and emus.

To assign studies to protected areas, we used the UNEP‐WCMC and IUCN Protected Planet: World Database on Protected Areas and World Database on Other Effective Area‐based Conservation Measures (UNEP‐WCMC and IUCN, 2023). The geographic coordinates for studies were either extracted from data presented in the publication (138 studies) or derived from Google Earth based on locations mentioned in the publication methodologies (24 studies). We used the *wdpar* package (v1.3.7; Hanson, 2022) in R to compare study locations to the Protected Areas map (UNEP‐WCMC and IUCN, 2023). The study was defined as being undertaken in a protected area if the animal was recorded within a protected area (i.e. national park or reserve) at any point during the study. The creation date of each protected area was compared with the study date to ensure that these overlapped before assigning protective status. Each study site was categorised by biome using the *readOGR* function in the *rgdal* R package (v1.6‐2; Bivand et al., 2022); these data were derived from Ecoregion Snapshots: Descriptive Abstracts of the Terrestrial Ecoregions of the World. 2021, developed by One Earth and RESOLVE. Version 2021 (www.oneearth.org). The global distribution of studies was mapped. Temperate climates are described as being >35° or < −35° N and tropical climates between 23.4° and −23.4° N. For each of the studies that had geographic location data, the Human Footprint Index (HFI) value was extracted from Venter et al. (2016) using the *extract* function in the *raster* R package (v3.6‐3; Hijmans, 2022). The coordinates used were the same as those used to assign protected status. HFI was used to identify the impact of human activity and landscape modification on seed dispersal. Two studies, Weir and Corlett (2007), and Wotton and Kelly (2012), were omitted from the seed dispersal analysis as there was no HFI data available for these locations. The HFI values extracted here use data from 2009 and do not align exactly with the dates of our studies estimating seed dispersal distances (ranging from 1987 to 2022), but we use these values as a broad‐scale indicator of human disturbance.

### 2.3 Data analysis

#### 2.3.1 Quantity and quality of tracking data

A total of 162 peer‐reviewed studies were included in this review, from which 107 were used to assess data quality and quantity. Twenty‐two studies were omitted because they tracked taxa with insufficient sample sizes for analysis (reptile = six studies & fish = two studies) or seed/fruit resources (14 studies). Resource tracking studies were omitted from this part of the analysis as this method of tracking is limited by study design and results were not comparable to the use of GPS and radio transmitters. A further 33 studies were omitted as the publications failed to report the required information, that is, individual tag performance and information was not recorded or available.

To calculate the mean number of tag days, 84 studies representing 130 individual species were used (see Table S2). To calculate mean tag locations, 81 studies representing 117 individual species were used (see Table S3). Generalised linear models (GLMs), with a gamma distribution and log‐link function, were fitted to assess the effect of taxa (bird or mammal) or tracking method (GPS or radio tracking) on the number of tagging days and number of tagging locations recorded. Models with and without interaction terms were compared using Akaike Information Criterion (AIC) values, and the model with the lowest AIC was selected.

#### 2.3.2 Likelihood of GPS tags being used

A total of 140 studies were used to assess whether the type of tracking method used could be predicted by taxa, body mass of species and year. Resource tracking studies and reptile and fish species were omitted. We fit binomial GLMs to the outcome of studies using GPS tags with taxa (bird or mammal), body mass (g) and year of study (1978–2022) as fixed effects. Models with and without interaction terms were compared using Akaike Information Criterion (AIC) values, and the model with the lowest AIC was selected.

#### 2.3.3 Drivers of seed dispersal distances

Finally, 67 studies calculated seed dispersal distances. We used 45 of these to assess mean seed dispersal distances and 56 to assess maximum seed dispersal distances. This information came from 61 and 71 individual animal species, respectively (see Appendix S3). Six studies reported median dispersal distances rather than mean. As these were the minority, we decided to remove these from the mean dispersal analysis. A further two studies were removed from both the mean and maximum dispersal analysis as these focused on species during their migrations (Mallard duck, Red‐billed teal & Egyptian goose) and the distances estimated did not allow for models to converge. As per previous analysis, studies focussing on fish or reptiles were also omitted because the number of studies were too small.

To assess the effect of body mass and different environmental variables on seed dispersal distances, we fitted GLMs, with a Gamma distribution and log‐link function, comparing the predictor variables of body mass, protected areas, volant/non‐volant, biome and the study site HFI score. We used volant/non‐volant in models rather than taxa due to collinearity between these two variables and because it provides greater functional information about the species than taxa alone. We did not include tracking method because of the limitations inherent to different methods, for example, longer dispersal distances are often identified from GPS tags due to remote download capabilities, while resource tracking is often limited to certain species, in particular small sized mammals. Body mass and HFI were scaled and centred around the means to ensure that they were comparable. To explore the impact of SRT and body mass on dispersal distances we explored the relationship between these two variables by firstly, calculating the Spearman's rank correlation coefficient to determine correlation and then fitting a linear model (LM) to determine the overall effect of body mass on SRT.

Statistical assumptions for each GLM and LM were validated by visual interpretation of residual diagnostic plots to check for linearity of model‐fitted values and their residuals. For each analysis, link functions were tested to determine the best residual distribution model based on AIC comparison and visual analysis of quantile–quantile plots of produced residuals using the *plot.DHARMa()* function in the *DHARMa* package (v0.4.6; Hartig, 2022). For the seed dispersal GLMs, a gamma distribution and a log link function allowed for the best model fit. The *dredge* function in the *MuMIn* package (v1.47.1; Bartoń, 2022) was used to assess the optimum variables for each model.

All analyses were performed using R Statistical Software (v4.2.2; R Core Team, 2021). A full summary of the different study subsets for each analysis is available in Table S12.

## 3 RESULTS

### 3.1 Literature review

We reviewed 162 peer‐reviewed research studies that used either radio transmitters or GPS tags to record the locations and movements of frugivorous animals. Of these 162 studies, 148 tracked frugivorous animals and 14 tracked seeds or fruits directly (see Table S13). Most of these studies focused on animal ecology and behaviour, and included themes such as *competition*, *behaviour*, *foraging ecology*, *habitat use*, *landscape connectivity*, *migration*, *movement ecology* and/ or *resource selection*, whereas 40 studies focused primarily on *plant ecology*, including *seed dispersal* and *plant recruitment*. The 162 articles identified in our search were published in 70 different journals covering different scientific themes, but principally *animal ecology*, *conservation biology* and *tropical ecology*. The most common journal was *Biotropica*, which published 11% of the studies reviewed here (see Appendix S4).

**FIGURE 1:**
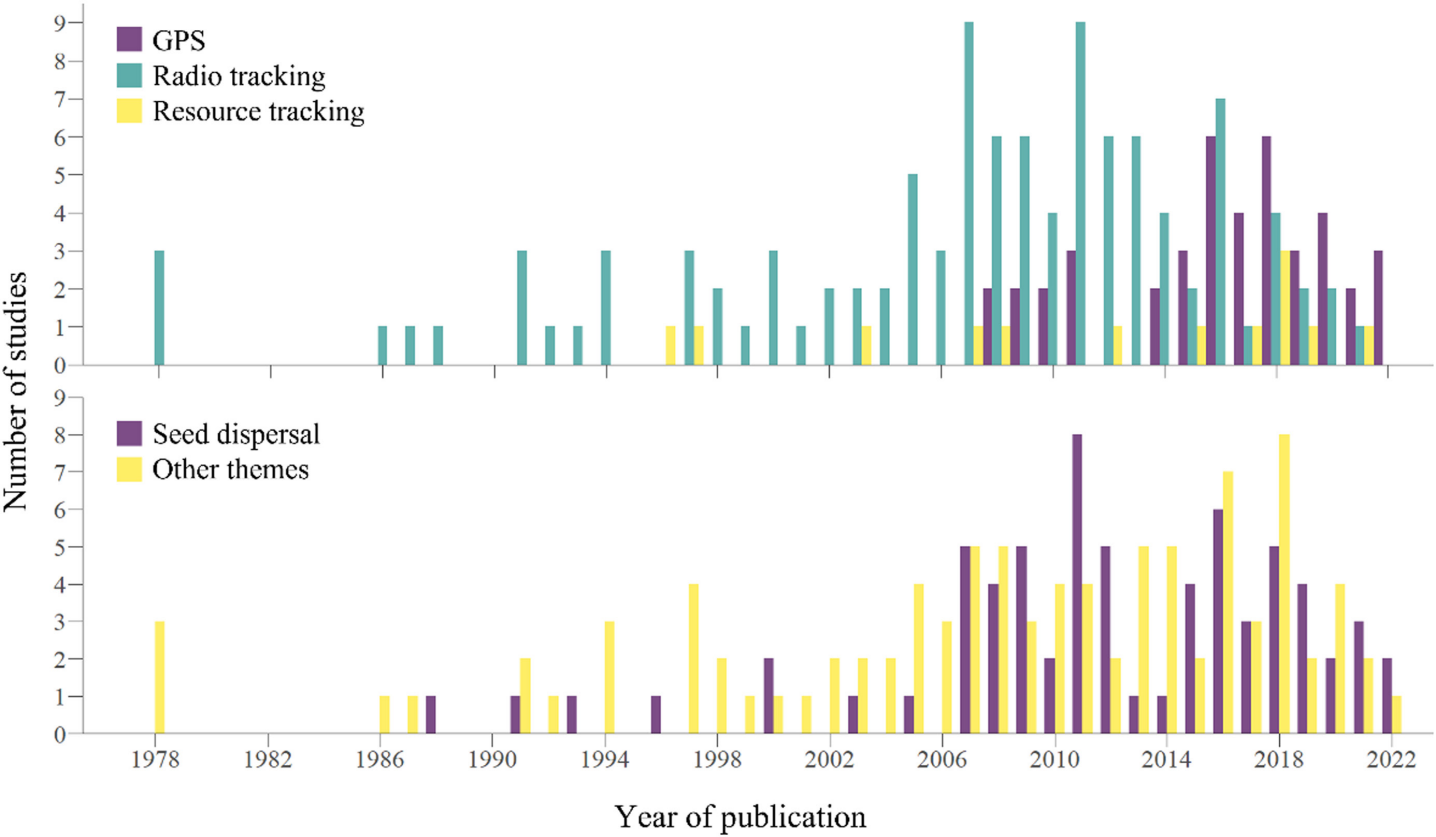
The number of published studies per years that used tracking technology (GPS, radio transmitters or resource tracking) on frugivorous species (a) and the number of published studies per years that either calculated seed dispersal distances or focussed on other themes (b) between 1978 and 2022.

The number of studies tracking frugivores increased from the first studies in the late 1970s to a peak of 13 per year in 2016 and 2018 (Figure 1). The earliest studies to use remote tracking techniques for monitoring frugivores were conducted in 1978 (see Heithaus & Fleming, 1978; Morrison, 1978a, 1978b), but it was not until 2005 that more than three studies were published per year (Figure 1). The first studies to track frugivores using GPS tags were undertaken in 2008, and both focused on elephants (see Blake et al., 2008; Campos‐Arceiz et al., 2008; Figure 1). Since then, the use of GPS tags in frugivore monitoring studies has increased, and from 2017, GPS tracking tags became more commonly used than radio transmitters (Figure 1).

The number of studies that estimate seed dispersal distances from tracking data also increased since the late 1970s to the present (Figure 1); the earliest study was published in 1988 (see Murray, 1988), but not until 2007 were more than two studies published annually (Figure 1). Eight studies were published 1988–2006, while 59 were published 2007–2022 (Figure 1); a 738% increase in the last 16 years compared to the previous 19 years.

### 3.2 Global distribution of tracking studies

**FIGURE 2:**
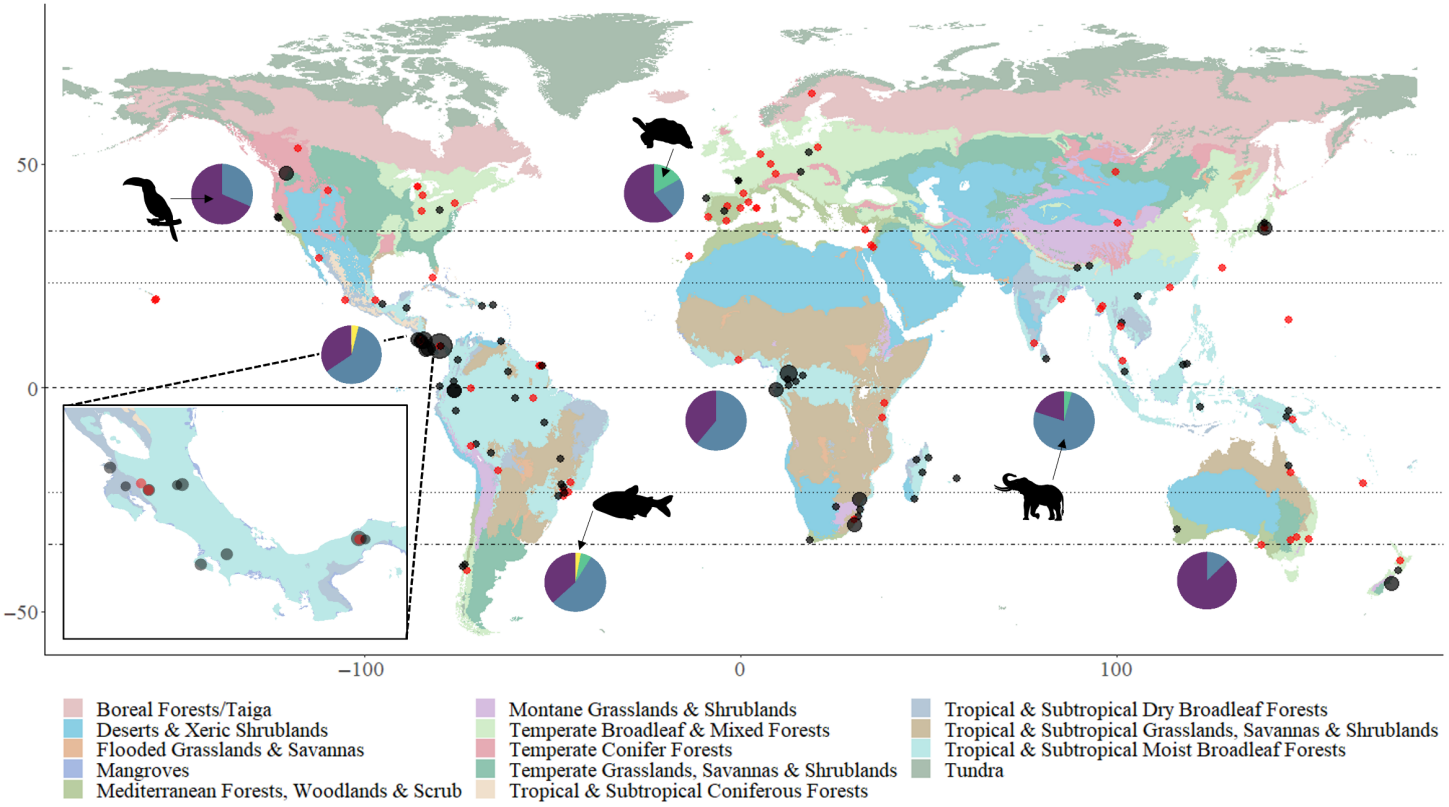
The geographic distribution of 162 frugivore tracking publications undertaken between 1978 and 2022. Number of studies per location are denoted by point size, with black points denoting studies undertaken inside protected areas, and red outside protected areas. Corresponding pie charts show the proportion of focal taxa represented in studies from each continent (Purple = Bird; Blue = Mammal; Green = Reptile; Yellow = Fish). Map colours represent the 14 biomes taken from *Ecoregion Snapshots: Descriptive Abstracts of the Terrestrial Ecoregions of the World. 2021*. Developed by One Earth and RESOLVE. Version 2021 <www.oneearth.org>. Downloaded on [April 2021].

Overall, 49 countries across six continents are represented in the 162 studies. Studies were not evenly distributed among countries, and 51 studies were conducted in just four countries: Costa Rica (9% of studies), Brazil (8%), USA (8%) and Panama (6%) (Figure 2). More studies were conducted in tropical and sub‐tropical than in temperate regions; this is illustrated in the two most well‐studied taxonomic group birds (studies in tropical/sub‐tropical regions 64% vs. temperate 36%) and mammals (tropical/sub‐tropical regions 83% vs. temperate 17%).

The number of studies conducted within and outside protected areas varied by region. The percentage of studies conducted within protected areas was highest in Central America and lowest in Europe (see Appendix S2; Figure 2). This is reflected in the research sites used in Central American studies; nine studies from Panama and 13 studies from Costa Rica were in protected areas. The proportion of studies that addressed the ability of species to disperse seeds also differed among regions. For example, 50% of all tracking studies but only 27% of all seed dispersal, were conducted in the Americas (see Appendix S2; Figure 2).

### 3.3 Focal taxa of tracking studies

Across studies, 165 species were tracked in four taxonomic groups: 84 bird species, 75 mammal species, four reptile species and two fish species. Almost half of the mammals (48%) studied were bat or flying fox species. Mammals were the most well studied group (49.4% of studies), followed by birds (45.7%), reptiles (3.7%) and fish (1.2%). Geographically, birds were the target of most tracking studies in Europe (61.1%), North America (68.4%) and Oceania (87.5%), whereas mammals made up most studies in tropical regions across South America (52.9%), Central America (61.5%), Africa (64%) and Asia (75%) (Figure 2). Only 48 species were studied more than once, including 23 birds, 23 mammals and 2 reptiles. The most common frugivores studied were the Seba's short‐tailed bat (*Carollia perspicillata*), one of the focal species of six studies, followed by the Eurasian Jay (*Garrulus glandarius*), African bush elephant (*Loxodonta africana*), Asian elephant (*Elephas maximus*) and Little yellow‐shouldered bat (*Sturnira lilium*), all tracked in four studies.

A diverse range of species were studied, which is shown by large ranges in body mass (7.6–44,000 g and 9.4–4,750,000 g, for birds and mammals respectively, see Table S1). Body mass proved significant in determining the likelihood of GPS tags being preferred for use in frugivore studies; with every one unit increase in body mass (g), there is an estimated 0.000012 increase in the log‐odds of GPS tags being used (estimate ± std error = 0.000012 ± 0.0000059, *p* = .0384, df = 195). However, there was no effect of taxa (estimate ± std error = 0.5365 ± 0.3577, *p* = .1337, df = 195) or an interaction between taxa and body mass (estimate ± std error = −0.000038 ± 0.000038, *p* = .321, df = 194) on the outcome of GPS tags being used (see Table S4b).

**FIGURE 3:**
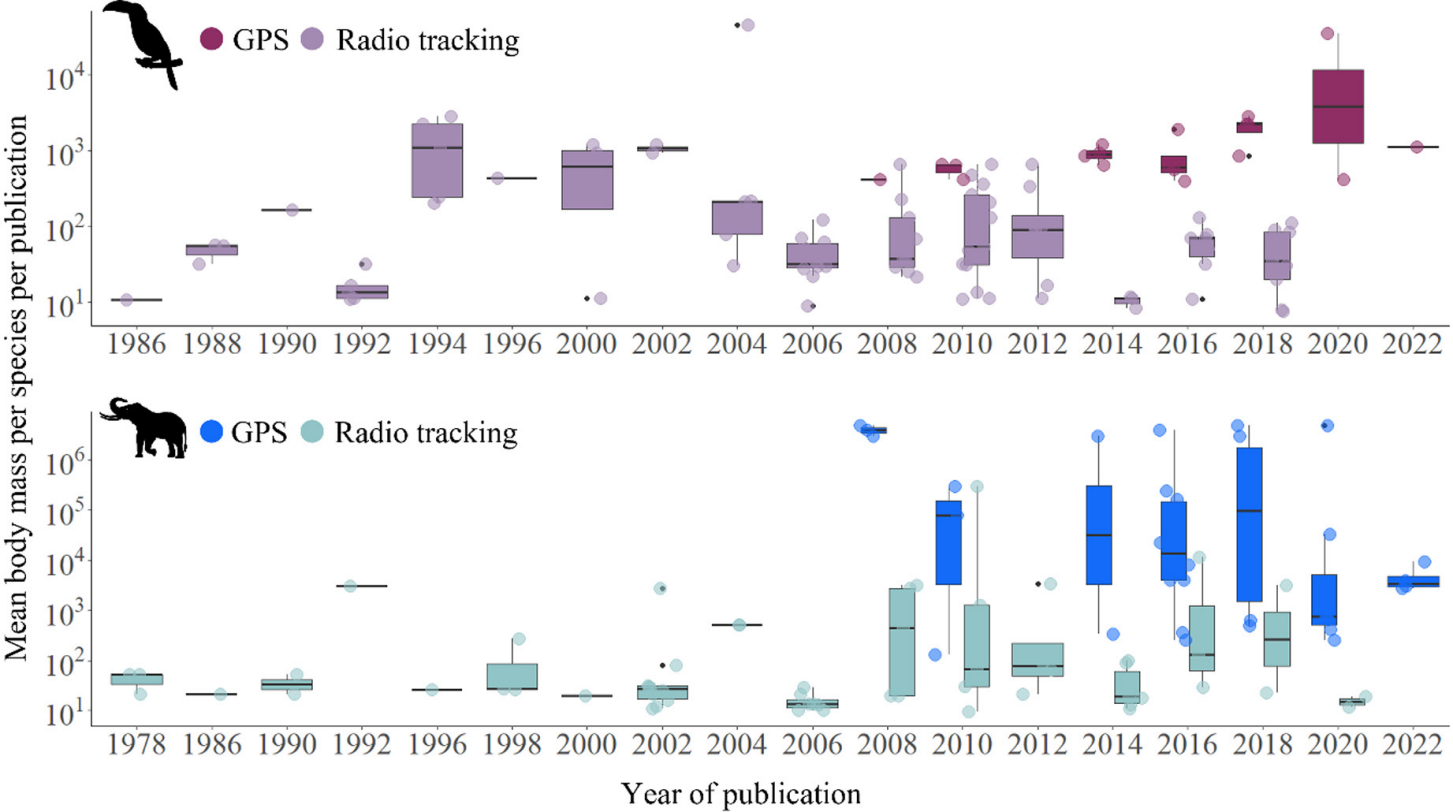
The mean body mass of species tagged, with either GPS tags or radio transmitters, per study during 1978–2022, for frugivorous birds, and frugivorous mammals.

For mammals, there was a significant effect of study year on the likelihood of GPS tags being used; with every succeeding year, there was an estimated expected 0.385 increase in the log‐odds of GPS tags being used (estimate ± std error = 0.3849 ± 0.09692, *p* = .000072, df = 89; Figure 3). However, body mass had no detectable effect (estimate ± std error = −0.000079 ± 0.000055, *p* = .147, df = 89), nor was there any detectable interaction between year and body mass (estimate ± std error = 0.0000027 ± 0.0000018, *p* = .125, df = 89, see Table S6b). For birds, there was no detectable effect of year on the outcome of GPS tags being used (estimate ± std error = −0.03877 ± 0.1432, *p* = .78666, df = 101). However, body mass (estimate ± std error = −0.03935 ± 0.01125, *p* = .00072, df = 101), and an interaction between body mass and year (estimate ± std error = 0.00146 ± 0.000412, *p* = .0.000408, df = 101; Figure 3) was significant in predicting the outcome of GPS tags being used (see Table S5b).

### 3.4 Quantity and quality of data collected

Among all studies analysed, 107 studies used radio transmitters, 42 used GPS and 14 studies tracked resources using radio transmitters and/or GPS tags (i.e. attached to seeds/fruits rather than individuals). One study used both radio and GPS tags to follow animals (Campos‐Arceiz et al., 2008; Asian tapirs). The mean number of tags deployed per species per study was 17.48 ± 1.67, the median 10, the range 1–172 and *n* = 206 (see Appendix S1). A total of 115 out of 148 animal tracking studies reported information necessary to calculate deployment successes. Of these, 49 studies (33.1%) recorded tag failure (tag loss, battery failure, insufficient data for analysis etc.), whereas 66 studies reported a 100% tag success rate. The average tag success rate across all studies was 86.2%. The tag success rate may be lower than reported as the remaining 33 studies did not clearly state whether the figures reported were the number of tags deployed or the number successfully returned and used in analysis.

**FIGURE 4:**
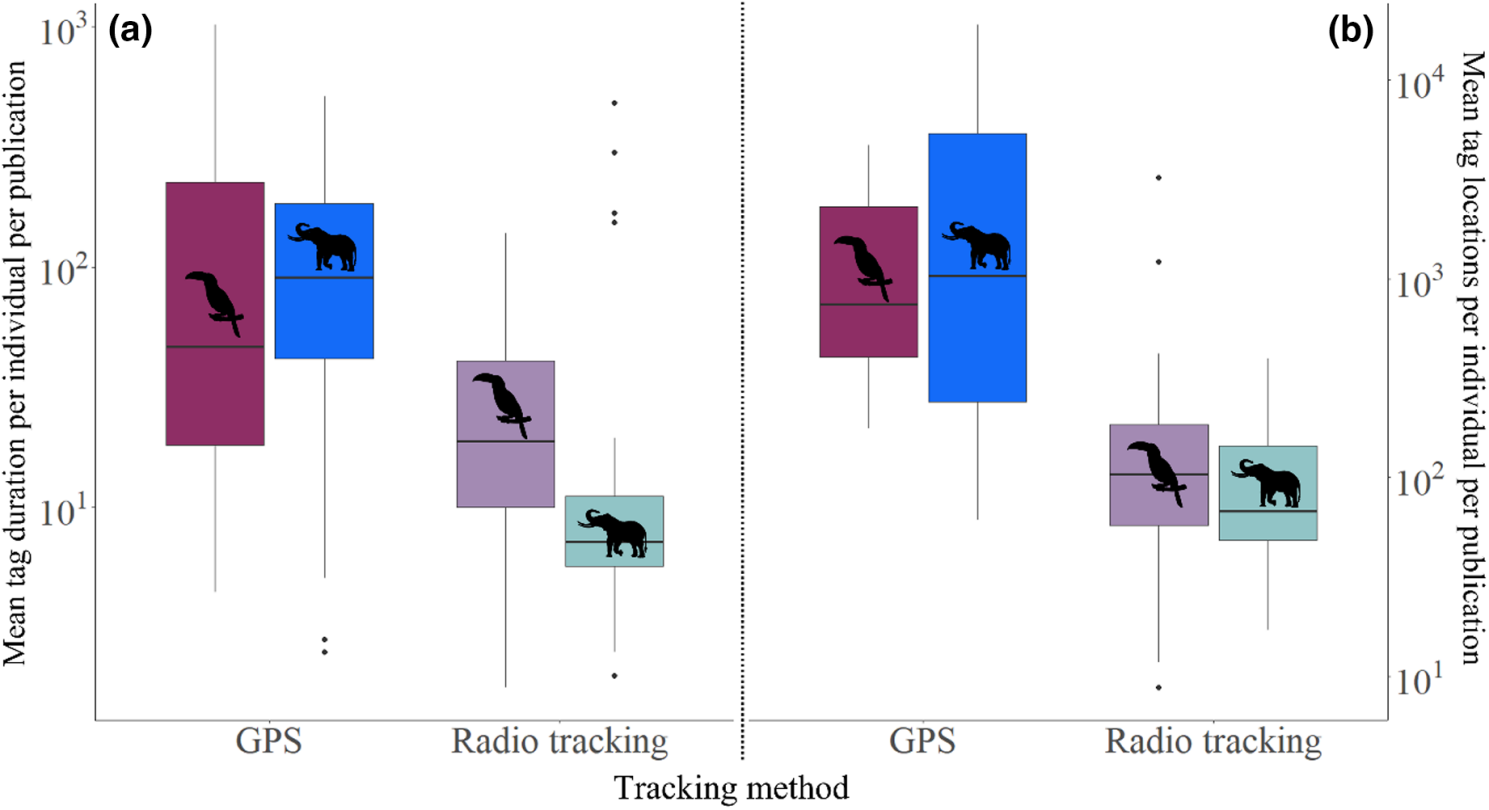
A comparison between tracking method (GPS & radio transmitters) and animal taxa (bird and mammal) with respect to (a) the mean number of tagging days and (b) the mean number of total location points collected per individual species per study.

Generalised linear models indicated that tracking method was a significant predictor of the duration of tags and the number of locations recorded per study (Figure 4). Studies using radio tags recorded fewer days (estimate ± std. error = −1.79 ± 0.49, *p* < .001; Figure 4a, see Table S7b), and locations (estimate ± std. error = −2.01 ± 0.48, *p* < .001; Figure 4b, see Table S8b), than GPS tags. Neither taxa nor an interaction effect between taxa and tracking method influenced duration of deployment, but there was an interaction effect between these two for the number of locations collected (estimate ± std. error = −1.58 ± 0.67, *p* < .05; Figure 4b).

### 3.5 Drivers of seed dispersal distances

**FIGURE 5:**
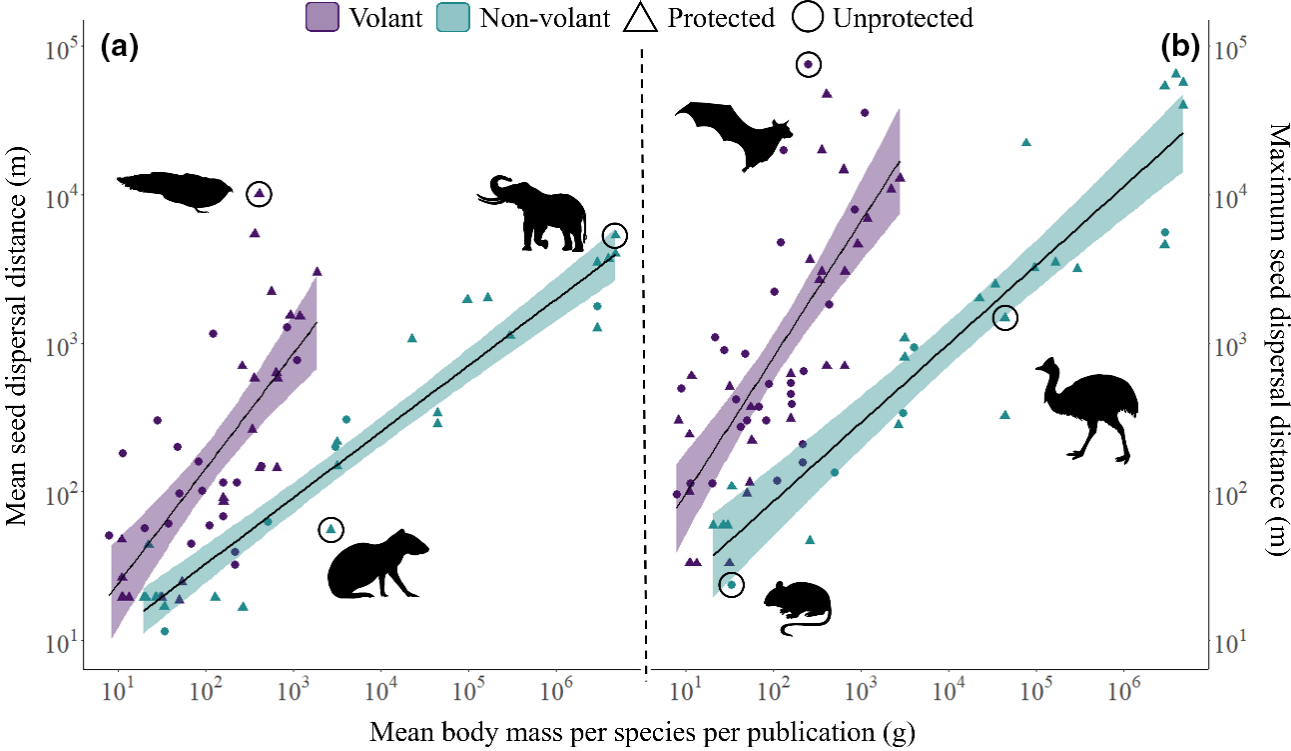
The estimated (a) mean and (b) maximum seed dispersal distances of bird and mammal species in relation to their body mass. Regression lines for volant/non‐volant species are denoted by colour and show standard errors with a 95% confidence interval. Point shape denotes whether the study was conducted in a protected area or not. Images indicate the species for the closest circled point and from left to right indicate Oilbird (*Steatornis caripensis*), Central American agouti (*Dasyprocta punctata*), African forest elephant (*Loxodonta cyclotis*), Large Japanese field mouse (*Apodemus speciosus*), Straw‐coloured fruit bat (*Eidolon helvum*) and Southern cassowary (*Casuarius casuarius*).

Mean and maximum dispersal distance increased significantly with species body mass (mean dispersal estimate ± std. error = 0.67 m ± 0.21 m, *p* = .00245; maximum dispersal estimate ± std. error = 0.78 m ± 0.28 m, *p* = .0071; Figure 5a,b). Volant species (bats, flying foxes and flying birds) had significantly higher mean seed dispersal distances than nonvolant species (mean dispersal estimate ± std. error = 0.95 m ± 0.45 m, *p* = .0384; Figure 5a). Volant species also had marginally significant higher maximum seed dispersal distances than nonvolant species (maximum dispersal estimate ± std. error = 1.02 m ± 0.6 m, *p* = .094; Figure 5b). Maximum dispersal distances also increased significantly with an interaction between body mass and volant species (maximum dispersal estimate ± std. error = 2322.85 m ± 726.28 m, *p* = .002; Figure 5b). The studies that were undertaken in protected areas also showed a significantly higher mean seed dispersal distance than those that were not in protected areas (mean dispersal estimate ± std. error = 1.29 m ± 0.43 m, *p* = .0037; Figure 5a), but these were not significant for maximum seed dispersal distances (maximum dispersal estimate ± std. error = 0.243 m ± 0.63 m, *p* = .7). Additionally, HFI was not significant for mean or maximum dispersal models and was not included in subsequent models (mean dispersal estimate ± std. error = 0.07 m ± 0.22 m, *p* = .7; maximum dispersal estimate ± std. error = 0.09 m ± 0.32 m, *p* = .8). Lastly, species with a larger body mass had a significantly longer seed retention time than smaller sized species, with SRT increasing by 30 seconds for every additional kilogram in body mass (Spearman's rho = 0.789, *p* = 9.52e−15, *n* = 64; mean seed retention time estimate ± std. error = 0.00054 min ± 0.0001 min, *p* = 1.57e−6; Figure S1) See Tables S9c, S10c and S11 for full model outputs.

## 4 DISCUSSION

### 4.1 Has there been a shift in the number of frugivorous tracking studies?

Seed dispersal and movement ecology are increasingly integrated, and the use of tracking technology to study frugivorous species has significantly increased in the past 17 years, from 33 studies between 1978 and 2004 to 129 studies from 2005–2022. The number of studies calculating seed dispersal distances from frugivore movement increased from 8 studies in 1988–2006 to 59 studies from 2007–2022 (Figure 1).

Radio tracking was the most commonly used tracking method for frugivores up until 2017, but since then, GPS tags have become the most used method. These increases are likely associated with the advancement in tracking technology, and specifically the reduced costs and increased availability of tags (Kays et al., 2015; Pimm et al., 2015). The smallest commercially available GPS tag in 2006 was 9.5 g (Microwave Telemetry Inc, 2021) whereas now tags weighing <3 g are commercially available from numerous companies (e‐obs GmbH, 2021; TechnoSmart, 2021). In addition, an increasing number of interdisciplinary teams are constructing GPS tags using commercially available components (Allan et al., 2013; Fischer et al., 2018; Paden & Andrews, 2020). These tags tend to be cheaper than traditional tracking devices, potentially increasing the number of research studies that have access to tracking technology. The potential for remote data download, ongoing battery miniaturisation and implementing solar‐powered recharge capabilities have enhanced our ability to monitor species across greater distances, in remote locations, and across diverse landscapes, where previous attempts failed (Bouten et al., 2013; Hart et al., 2020; Shimada et al., 2020).

### 4.2 Our current understanding of frugivorous tracking studies

#### 4.2.1 Global distribution

Over half (59.3%) of all frugivore tracking studies (162 studies) reviewed here were in tropical regions (between 23.436° N and 23.436° S; Figure 2), a further 14.2% were from the sub‐tropics, and 26.5% were from the southern and northern temperate zones. This reflects latitudinal patterns of higher species richness in tropical compared to temperate regions. However, this pattern was not seen in studies that calculated seed dispersal distances (subset of 67 studies), where only 31% of studies were in tropical regions, and does not reflect difference in animal‐mediated seed dispersal globally; up to 60% of temperate plants rely on animal dispersal compared to 90% of tropical plants (Gentry, 1988; Howe & Smallwood, 1982). This suggests that in the tropics we see a lack of seed dispersal studies using animal movement data, potentially because fieldwork locations and dense forests provide challenges to fieldwork and transmitters (Kays et al., 2011; Monsieurs et al., 2017). However, the uptake of solar powered tags, improvement in battery capacity and remote downloading capabilities may soon rectify this (Byers et al., 2017; Fischer et al., 2018). Our data also suggest that many regions have yet to be explored for the study of frugivore movement, for example Eastern Europe, Central & Northern Africa, Central Asia and much of North America lack tracking studies (Figure 2).

Sites where multiple studies have been conducted tended to be in protected areas and/ or at key field stations. In total, 172 different locations were used for these tracking studies, with almost two thirds (59%) in protected areas, with clustering of multiple studies at long‐term research stations (Blanco et al., 2020). This is both a benefit and a limitation; on the one hand, highly studied sites become hotspots for research, with multiple taxa studied in a single location and often with longitudinal datasets (Stouffer, 2020). This provides information on how multiple taxa respond to the same landscape changes and how patterns may differ among species. Alternatively, concentrating multiple studies in a few locations means that we make inferences from a handful of intensely studied sites and lack broader knowledge from diverse locations and landscapes. Most studies we reviewed were in protected areas, but these represent ~17% of terrestrial land surface area (UNEP‐WCMC and IUCN, 2023), suggesting that we need further studies outside parks and reserves as well as comparative studies in protected areas and neighbouring disturbed habitats to effectively survey a representative sample of habitats.

#### 4.2.2 Focal taxa

Broadly equivalent numbers of studies focused on birds and mammals (74 and 80 respectively). In total, 29% of species were studied more than once, and the most heavily studied species were frugivores that are known to be readily caught and tagged, and those commonly found in research hotspots (Biro, 2013; Rosenthal et al., 2017). These frequently studied species suggest that frugivore tracking studies focus on key long‐distance dispersers, including larger‐bodied animals that can disperse larger seeds across greater distances (African bush elephant & Asian elephant) and animals that have the ability to fly and connect fragmented landscapes (Little yellow‐shouldered bat and Seba's short‐tailed bat).

Bats were the most intensely studied group of mammals: a total of 40 studies and half of the mammal studies. The high number of bat studies represents the high diversity of frugivorous bats and their importance in long distance dispersal (Muscarella & Fleming, 2007). However, only four studies calculated seed dispersal distances, potentially due to tag weight limitations. Many bat species are too small to carry tracking devices (O'Mara et al., 2014; Van Harten et al., 2019), and those that are not, are often constrained to just a few weeks of data collection because of common tag attachment techniques, for example, surgical glue. While this method is widely used, tags rarely remain attached for longer than 4 weeks (O'Mara et al., 2014), and therefore only provide a limited snapshot of a species movement capabilities. Bats provide important links among forest fragments due to their mobility (Estrada & Coates‐Estrada, 2002), and with the advent of smaller tags (Dressler et al., 2016), future studies could better explore their role in seed dispersal.

Four species of reptiles were tracked across these studies including the yellow‐footed tortoise (*Chelonoidis denticulatus*), Lilford's wall lizard (*Podarcis lilfordi*), Eyed lizard (*Timon lepidus*) and Southeast Asian box turtle (*Cuora amboinensis*), suggesting that reptiles are currently being underrepresented in biologging studies in terms of their potential role as seed dispersers. This is of particular importance for island habitats where reptiles, predominately lizards and tortoises, often occur disproportionately compared to other species. These habitats are often species‐poor in terms of diversity, meaning reptiles become some of the only seed dispersers around (Olesen & Valido, 2003). In particular, giant tortoises are thought to fill traditional megaherbivore roles on islands and are noted as ecosystem engineers (Blake et al., 2012; Falcón et al., 2020). Therefore, future seed dispersal studies should be encouraged to quantify the role that reptiles play as seed dispersers.

Body mass is clearly instrumental in determining which tracking method is likely to be selected for frugivore studies; globally, 72% of bird species and 55% of mammal species weigh less than 100 g (Wilman et al., 2014), which is the minimum body mass for a 5 g tag (typical for commercial GPS tags; Altobelli et al., 2022). In our review, the median body mass was 83.4 and 192.8 g for birds and mammals, respectively, suggesting that larger animals in general have a significantly increased likelihood of GPS tags being deployed compared to smaller animals, irrelevant of taxa. While radio transmitters can weigh as little as 0.2 g (Naef‐Daenzer et al., 2005) and offer a low‐cost alternative to GPS tags, there are trade‐offs with the quality of data collected (Gottwald et al., 2019). Radio telemetry often results in low temporal and spatial resolution due to infrequent location fixes and the required intensive labour in collecting these fixes (Alexander & Maritz, 2015; Harris et al., 1990; Ryan et al., 2004), thus GPS tags are often preferred once an animal passes a minimum size requirement.

Since miniaturisation and technological advances have reduced the size and weight of GPS tracking technology, we would expect frugivores to be tagged with GPS tags more frequently in recent years, but we did not see a clear pattern. For mammals, we found that in later years there was an increased probability of GPS tags being deployed, but body mass did not have a significant effect. We observed the opposite effect with bird species, with increased body mass there was an increased probability of GPS tags being deployed whereas year alone had no effect. However, an interaction between body mass and years shows a significant effect on the increased probability of GPS tag being deployed for bird species.

The pattern seen here suggests to us that the technological advances made concerning increased storage and remote download capabilities of GPS tags (Kays et al., 2015) have led to the increase of studies focusing on large, frugivorous bird species that have migrational behaviour or extensive home ranges where previous use of radio transmitters would have been ineffective (Hallworth & Marra, 2015; Lenz et al., 2015).

#### 4.2.3 Assessing the differences in quantity and quality of data collected through tracking technology

To effectively describe the animal movement and behaviour critical to seed dispersal predictions, we need detailed tracking data. Large time gaps and short study durations can limit inference and may lead to over or underestimating dispersal distances. Both tag duration (*length of time attached to an individual*) and total location points (*number of locations collected per individual tag*) significantly related to the tracking method. Studies using GPS tags captured almost 18 times the number of locations compared to studies using radio tags and were deployed for almost 5 times longer. Increased battery capacity and solar powered tags enable data collection over many months and possibly years (Silva et al., 2017).

GPS tags are increasingly used to track frugivores; since 2015, 28 studies have used GPS tags compared to just 11 studies during the previous 36 years. This has also allowed for a larger diversity of species to be tracked. Before 2003, the largest frugivore equipped with a tracking device was 3 kg. Since then, a further 21 species of frugivore have been tagged, with body mass ranging from 3 to 4750 kg. This trend could be linked to the remote download capabilities of GPS tags. Larger animals tend to have larger home ranges (Harestad & Bunnel, 1979) and GPS tags can now be downloaded from many kilometres away or via remote upload to satellites or Wi‐Fi (Kays et al., 2015). This is particularly important for migratory animals, where it is often impossible to stay close enough to use radio transmitters (Guilford et al., 2011). With the introduction of remote downloading, GPS tags are preferred because data download is guaranteed after the initial device attachment, without the need to recapture individuals or search for radio transmissions. Additionally, many GPS tags also include the option of onboard accelerometers (Brown et al., 2013; Shepard et al., 2008), which can be used for defining specific behaviours, such as foraging events, that can be incredibly useful when determining seed shadows.

On the other hand, many bird species are still tracked with radio transmitters. Radio transmitters tend to be smaller and can be used on smaller species, but this result may also relate to habitat. Most tropical birds reside within thick vegetation (MacArthur & MacArthur, 1961). GPS tags require low vegetation cover for successful fixes. In dense vegetation, GPS fixes can fail or overestimate movement tracks for up to an additional 28% (DeCesare et al., 2005).

### 4.3 Can body mass and environmental variables be used as predictors for seed dispersal distances?

Frugivore body mass was a key predictor for seed dispersal distance; both mean and maximum seed dispersal distance are positively correlated to body mass in birds and mammals. Larger species tend to have larger foraging areas and range distributions and so carry seeds further from the parental source (Godínez‐Alvarez et al., 2020). Flying species also had larger mean distances than non‐flying species, suggesting that bird and bat species are key for long distance seed dispersal events (Medellin & Gaona, 1999). This is further strengthened by maximum seed dispersal distances significantly increasing with an interaction between a species body mass and ability to fly. This indicates that an increase in body mass of a volant species has a greater, positive effect on maximum dispersal distance compared to the same increase for a nonvolant species.

Most studies that calculated seed dispersal distances focussed on birds, which are key for seed dispersal due to their mobility and ability to cross matrices and connect habitat patches (Mueller et al., 2014). Additionally, there is large interspecific diversity among the functional traits of bird species, which offers a wider dietary scope and allows them to be relatively flexible to switch to other resources in response to fluctuations in fruit resource availability through seasonal or land use changes (Bender et al., 2017). Among the most frequently studied bird species were Eurasian jay (*Garrulus glandarius*), Clark's nutcracker (*Nucifraga columbiana*), white‐crowned manakin (*Pseudopipra pipra*) and Swainson's thrush (*Catharus ustulatus*) – these species have broad, omnivorous diets, ranging from insects to fruit. Birds with generalist diets are key to seed dispersal in fragmented landscapes and tend move between different habitats and facilitate early forest succession in open areas (Barros et al., 2019; Carlo & Morales, 2016).

Bats species, like birds, can functionally connect fragmented landscapes, and are associated with long‐distance seed dispersal (Abedi‐Lartey et al., 2016). Although bats are often smaller than birds, the four studies in our review that focused on bats reported dispersal distances that tended to be greater than distances commonly reported for birds of similar body mass (Egyptian fruit bat, *Rousettus aegyptiacus* – 132 g, Madagascan flying fox, *Pteropus rufus* – 361 g, Orii's flying fox, *Pteropus dasymallus inopinatus* – 435 g, Straw‐coloured fruit bat, *Eidolon helvum* – 253 g), with Straw‐coloured fruit bats travelling up to 70 km at night (Abedi‐Lartey et al., 2016).This is perhaps possible due to their gap crossing abilities, nocturnal activities, plasticity of habitat use, and foraging strategies (Lourie et al., 2021; Muscarella & Fleming, 2007; Regolin et al., 2020). Furthermore, bats can defecate during flight, as opposed to when perched like most birds, which increases the likelihood of seeds being deposited in open areas where pioneer plant species can recruit and initiate forest regeneration (Muscarella & Fleming, 2007; Peña‐Domene et al., 2014; Regolin et al., 2020). For instance, *Phyllostomid* bats can disperse seeds of over 300 plant species (Lobova et al., 2009; Voigt et al., 2017) and will regularly commute between foraging areas in natural and degraded landscapes, enabling establishment of early successional plant species (Galindo‐González et al., 2000; Ripperger et al., 2015). Seed‐handling by bats could also increase their effectiveness as long‐distance dispersers (Ong et al., 2022), since fruits taken by bats are not limited by mouth/beak gape width, and some bat species carry fruits that exceed their own body mass (Mahandran et al., 2018).

Much like seed‐handling, understanding seed retention time (the time between seed ingestion and defaecation) is crucial for the estimation of seed dispersal distances and identifying potential deposition sites of seeds. The duration a seed spends within an individual's digestive system directly influences the distance it will be transported before being deposited (Schupp et al., 2010). However, assessing species seed retention time can often be challenging and complex due to the variability within animal species and across different plant species, and their fruits and seeds (Côrtes & Uriarte, 2013). Fruit secondary compounds and ripeness have been found to influence dispersal distances by significantly decreasing or increasing SRT within frugivorous bats, depending on the plant species (Baldwin et al., 2020; Baldwin & Whitehead, 2015). Conducting captive trials is possible, but these can be biased as the fruits consumed are often not representative of the frugivore's diet and can even be seeds implanted in pulp of cultivated fruits (Holbrook & Loiselle, 2007; Kays et al., 2015; Qian et al., 2022).

Given these challenges, researchers often need to use a combination of approaches, including controlled experiments, field observations and modelling, to gain a comprehensive understanding of seed retention time in a particular animal species (Adams et al., 2022; Cousens et al., 2010; Yumoto et al., 1999). Despite these difficulties, obtaining accurate data on seed retention time is crucial for assessing the role of animals in seed dispersal and predicting their ecological impact. Commonly, researchers use allometric scaling of body mass to determine estimates for seed retention time per animal species (Yoshikawa et al., 2019). At the species level, we also found a tight relationship between body mass and SRT (Figure S1; Table S11a,b, however this approach obscures fine‐scale variation driven by diet choices and intraspecific variation among frugivores and we highlight the need for more detailed seed retention information.

Finally, our models also appear to show a trend in mean dispersal distance for those studies undertaken in protected areas. These areas are likely to offer large tracts of undisturbed, continuous habitat enabling frugivores are able to travel long distances unrestricted by inhospitable landscapes such as intensive farmland or urban areas. In our analysis, animals may have moved out of protected areas into surrounding disturbed habitat during the duration of the study. However, our results still support the idea that dispersal distances are longer in areas with more intact habitat and validates previous evidence that seed dispersal services are likely to reduce in areas of landscape change (Wright & Duber, 2001). Furthermore, protected areas ensure frugivore safety through reducing illegal hunting events. This has a direct, positive effect on frugivore abundance and population levels that can increase seed dispersal services (Beckman & Muller‐Landau, 2007; Nuñez‐Iturri & Howe, 2007; Wright et al., 2000).

### 4.4 Recommendations

We reiterate the following points from previous reviews (Barron et al., 2010; Bodey et al., 2018) that it would be beneficial to do the following: standardise terminology, report device type and attachment, and provide all collected data. Many publications are still not following the protocols suggested in these publications, which are vital for future improvements to studies and analysis and will ensure that comprehensive comparisons between studies and species can be undertaken. Reporting the necessary key information can help ensure that standard protocols are followed and therefore improved (Andrews et al., 2019).

To improve data management, accessibility, and analysis, we recommend standardising animal tracking terminology when referring to tracking technologies. Currently, multiple words and terms are used synonymously across publications (Cooke et al., 2021). We recommend using the terms “GPS tags/transmitters” and “radio tags/transmitters” opposed to “units” or “trackers”. Terms such as “radio telemetry” are also used as common synonyms, however this refers to the whole radio tracking system: a radio transmitter, a radio antenna and a radio receiver. The terms “biologging” and “data loggers” are often used as an umbrella term (Cooke et al., 2021; Whitford & Klimley, 2019), but should be used in conjunction with GPS or radio tags/transmitters so that readers are aware of the technology used. Units would ideally be used to describe the whole device being attached to an animal, which may include accelerometers and/or environmental recording devices.

Studies present multiple different sampling rates and durations, so almost all movement studies are unique. They are often driven by constraints on the number of individual tags that can be deployed, and ultimately rely on the restrictions of capturing animals to tag and the funding of different projects. However, with the improvement and reduced costs of GPS tags, it would be highly beneficial for the community to aim to develop some standard minimum sampling rates and durations to make diverse datasets more compatible (Campbell et al., 2016; Sequeira et al., 2021). Tag failure must also be considered in future studies, and it is critical that studies report all data including tag failures and discrepancies from methods and results.

Finally, to allow reproducibility and future analysis, movement data should be shared in global data repositories. Making data publicly available increases broad and inter‐disciplinary collaborations and ensure that data are used most effectively. Data can also be used for further analysis by other scientists from different academic backgrounds and facilitate greater interdisciplinarity between subjects and ensures that data are used to their maximum potential. Repositories can help safeguard fundamental baseline data, which helps drive broader, temporal ecological questions that would not be possible with single or few studies (Davidson et al., 2020; Rutz et al., 2020; Tucker et al., 2018). This is already beginning to happen with data repositories for animal tracking, such as Movebank.org, and through conversations regarding registering all tracking device deployments (Rutz, 2022).

## 5 CONCLUSION

Increases in tracking studies, coupled with the advancement of tracking technology, have led to an exponential increase in seed dispersal studies over the past 17 years, particularly those that estimate seed dispersal distances. This offers a step forward in understanding how changes to landscape structure, for example, from land‐use change, can affect plant colonisation and forest recovery through understanding the movement patterns and behaviours of frugivores through tracking.

We see the next step forward in future seed dispersal studies as straightforward: more studies and repetition. Long‐term tracking studies from diverse taxa are necessary to collect movement and behaviour information. Many current tracking studies are limited by battery consumption and tag memory and are simply capturing a small snapshot of an individual's life and do not consider how temporal changes (e.g. seasons, anomalous years) may affect movement. With longer‐duration and finer‐scale data, we can begin to understand the drivers of animal movement and the implications for seed dispersal and other ecosystem services in a changing world. Ultimately, seed dispersal distances can successfully inform restoration and conservation projects, but only if estimations are accurate. Only by tracking frugivores can we ensure that this transpires. Through an understanding of seed dispersal, local organisations can manage landscapes to increase the potential of between‐patch connectivity to encourage plant regeneration and gene flow.

## AUTHOR CONTRIBUTIONS

**Adam Fell:** Conceptualization (equal); data curation (lead); formal analysis (lead); funding acquisition (supporting); investigation (lead); methodology (lead); project administration (lead); resources (equal); software (equal); supervision (supporting); validation (supporting); visualization (lead); writing – original draft (lead); writing – review and editing (lead). **Thiago Silva:** Conceptualization (supporting); data curation (supporting); formal analysis (equal); funding acquisition (supporting); investigation (equal); methodology (supporting); project administration (supporting); resources (supporting); software (equal); supervision (equal); validation (equal); visualization (equal); writing – original draft (equal); writing – review and editing (equal). **A. Bradley Duthie:** Conceptualization (supporting); data curation (supporting); formal analysis (supporting); funding acquisition (supporting); investigation (supporting); methodology (supporting); project administration (supporting); resources (supporting); software (supporting); supervision (equal); validation (equal); visualization (equal); writing – original draft (equal); writing – review and editing (equal). **Daisy Dent:** Conceptualization (equal); data curation (equal); formal analysis (equal); funding acquisition (lead); investigation (equal); methodology (equal); project administration (equal); resources (equal); software (equal); supervision (equal); validation (equal); visualization (equal); writing – original draft (equal); writing – review and editing (equal).

## FUNDING INFORMATION

NERC IAPETUS2 Doctoral Training Programme – NE/s007431/1.

## CONFLICT OF INTEREST STATEMENT

The authors declare that they have no conflicts of interest regarding the research, authorship, and/or publication of this work. This includes financial interests, affiliations, or involvement with any organisation or entity that could influence the conduct or reporting of the research.

## Supporting information


Appendix S1

Appendix S2

Appendix S3

Appendix S4

## Data Availability

A list of the reviewed studies and the information collated from these studies for the analysis within this review: Supplementary Material and Table S13. All model outputs: Supplementary Material and Tables S4–S11.
