# Supplementary material for: A global systematic review of frugivorous animal tracking studies and the estimation of seed dispersal distances
Source: Ecol Evol. 2023 Oct 31;13(11):e10638. doi: 10.1002/ece3.10638 (PMC10616751; doi:10.1002/ece3.10638)
Supplement: Supplementary file 1 — Appendix S1 [file ECE3-13-e10638-s004.docx]

Supplementary material 1. Information collated from all 162 frugivorous tracking publications. Information includes, but is not limited to, species, weight, location, number of tags deployed and the quantity and quality of data collected.

Supplementary material 2. Information collated from the 67 frugivorous tracking publications which were used for estimating seed dispersal distances. Information includes, but is not limited to, species, weight, human footprint index, mean seed dispersal distance and maximum seed dispersal distance.

Supplementary material 3. A list of keywords associated with each of the publications in this study and the scientific journal in which each publication was published.

Supplementary figure 1. The mean body mass of volant and non-volant species in relation to their mean seed retention time (SRT). Data extracted from a total of 42 studies that calculated seed dispersal distances and covers 59 different animal species. SRT is an average over multiple seed types for each species for each study. Regression line for all species shows standard error with a 95% confidence interval.


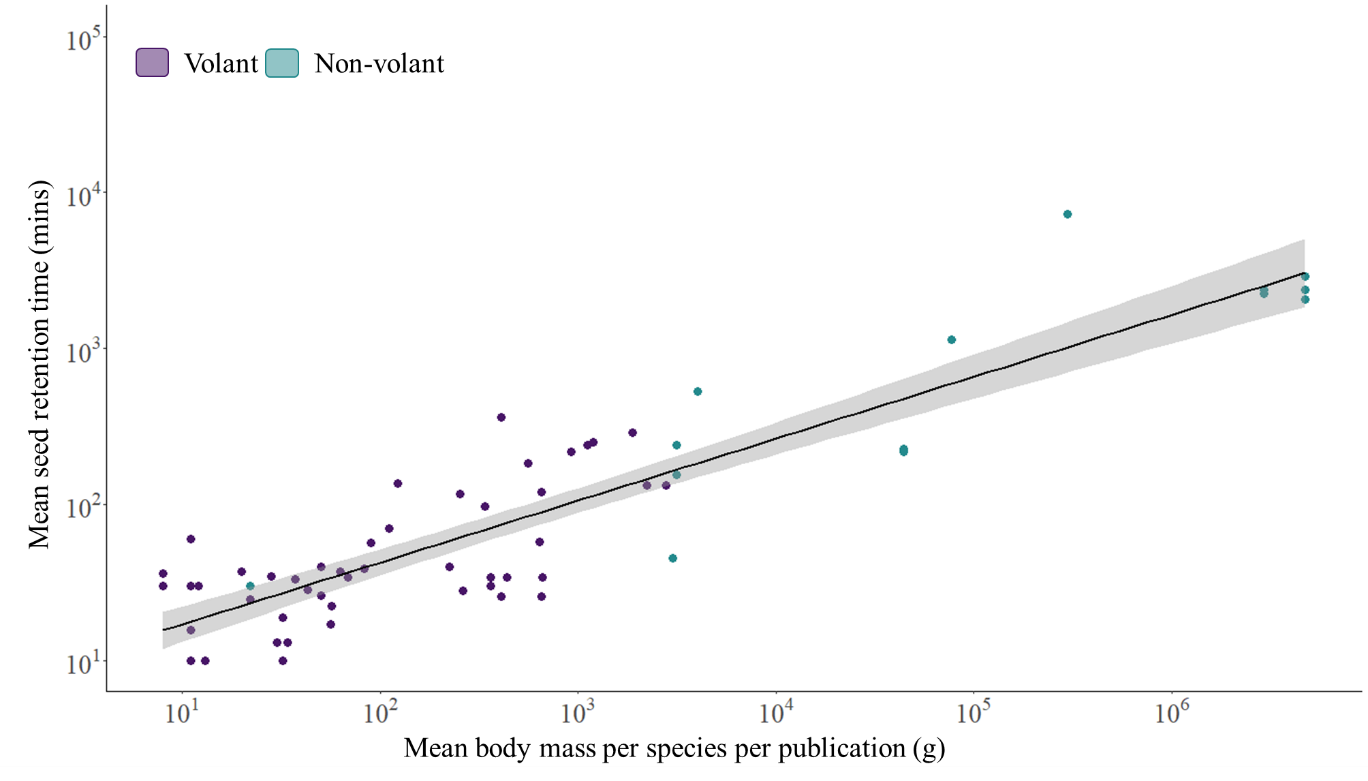


*Supplementary tables:*

Supplementary table 1. Summary statistics of the mass (g) of taxa tagged with different devices.


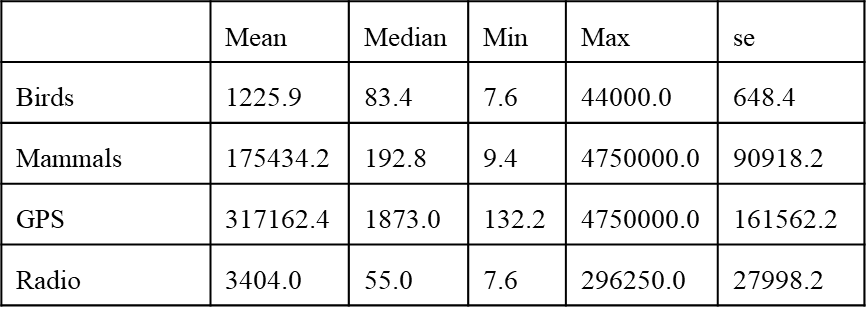


Supplementary table 2. Summary statistics for the number of days tags were deployed for on different taxa and for different devices.


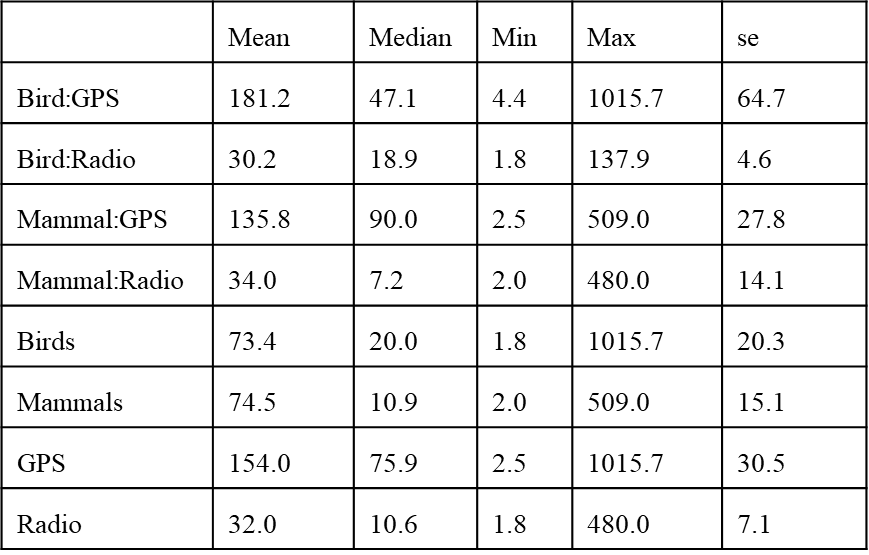


Supplementary table 3. Summary statistics for the number of locations collected on different taxa and for different devices.


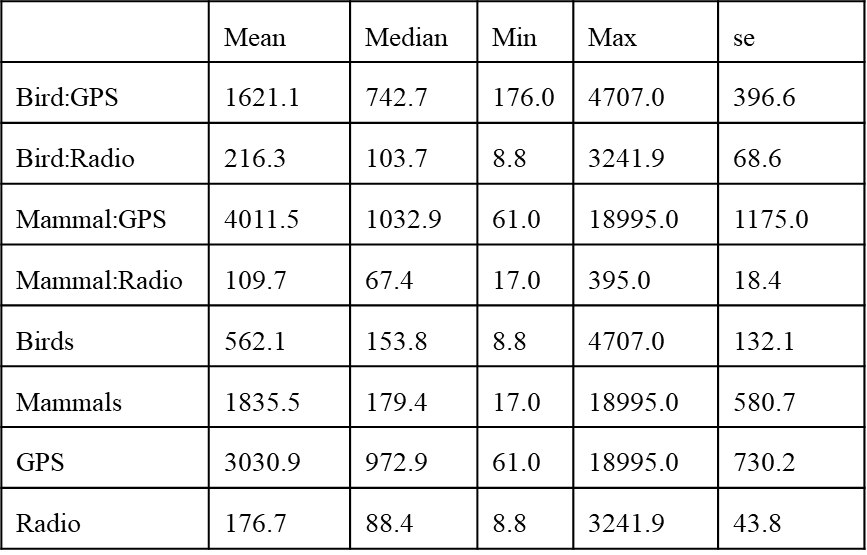


Supplementary table 4a and 4b. 4a) AIC scores to determine the best model for predicting tracking method GLM with body mass and taxa as predictors. 4b) Coefficient output table for predicting tracking method GLM with body mass and taxa as predictors.


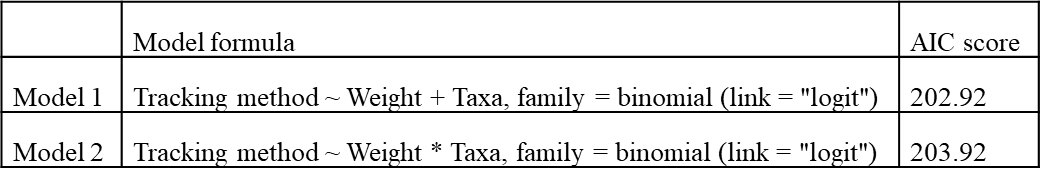


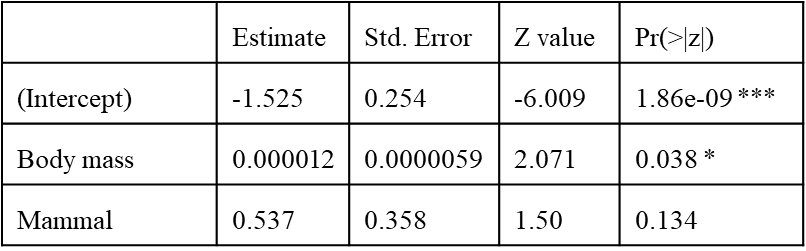


Supplementary table 5a and 5b. 5a) AIC scores to determine the best model for predicting tracking method GLM with body mass and year as predictors for the subset of bird species. 5b) Coefficient output table for predicting tracking method GLM with body mass, year, and an interaction term as predictors for the subset of bird species.


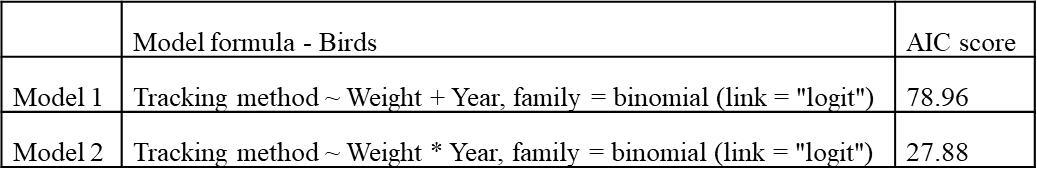


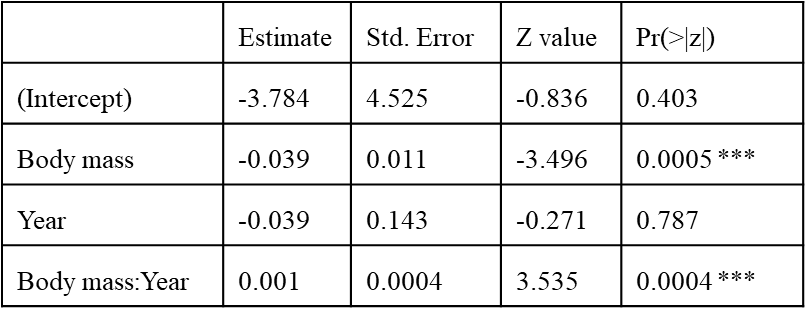


Supplementary table 6a and 6b. 6a) AIC scores to determine the best model for predicting tracking method GLM with body mass and year as predictors for the subset of mammal species. 6b) Coefficient output table for predicting tracking method GLM with body mass, year, and an interaction term as predictors for the subset of mammal species.


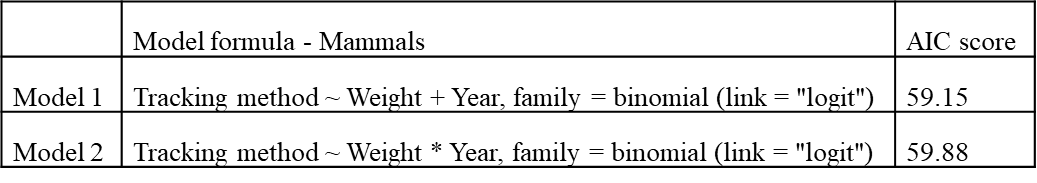


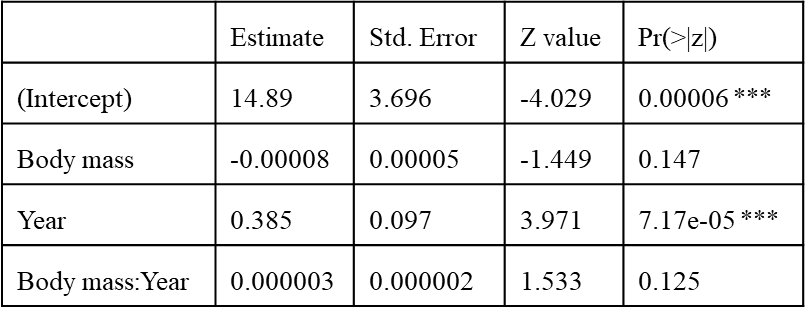


Supplementary table 7a and 7b. 7a) AIC scores to determine the best model for predicting the number of tracking days GLM with tracking method and taxa as predictors. 7b) Coefficient output table for predicting the number of tracking days GLM with tracking method, taxa, and an interaction between the two as predictors.


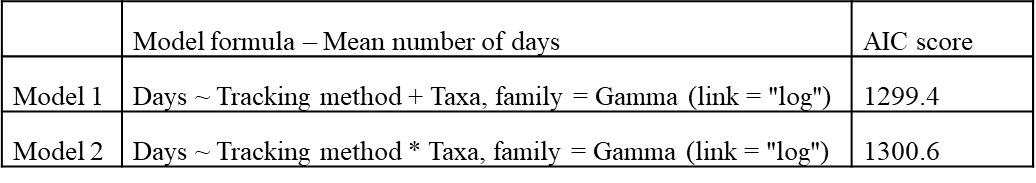


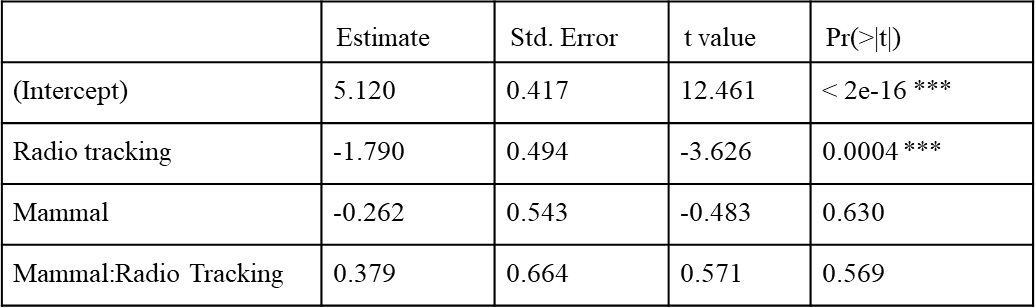


Supplementary table 8a and 8b. 8a) AIC scores to determine the best model for predicting the number of tracking locations GLM with tracking method and taxa as predictors. 8b) Coefficient output table for predicting the number of tracking locations GLM with tracking method, taxa, and an interaction between the two as predictors.


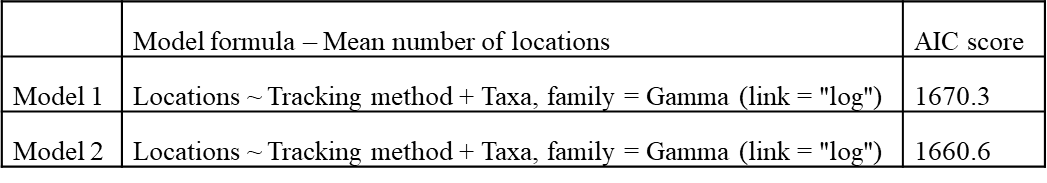


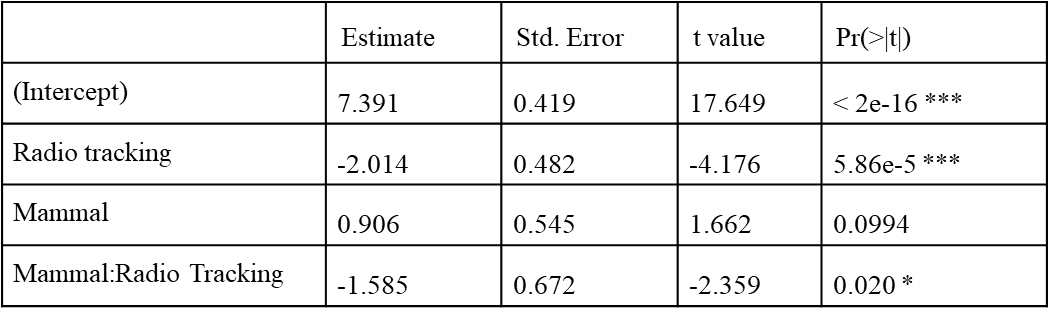


Supplementary table 9a, 9b and 9c. 9a) Model selection output for the best fit model for mean seed dispersal distance GLM with body mass, volant, protected and HFI as predictors. 9b) AIC scores to determine the best model, with or without an interaction, for predicting mean seed dispersal distance GLM with body mass, volant and protected as predictors. 9c) Coefficient output table for mean seed dispersal distance GLM with body mass, volant and protected areas as predictors.


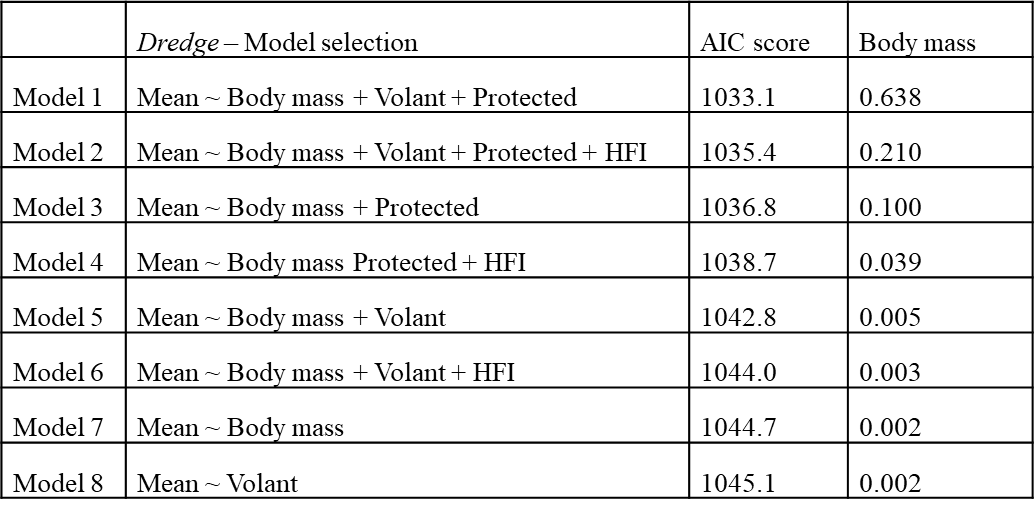


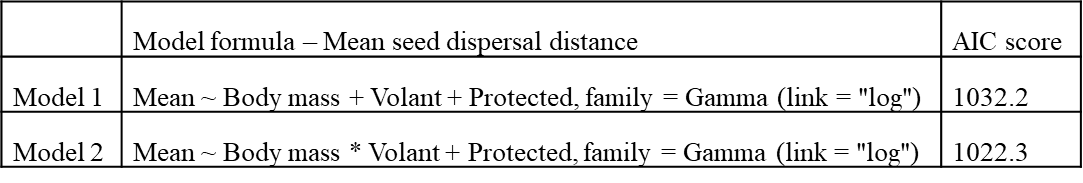


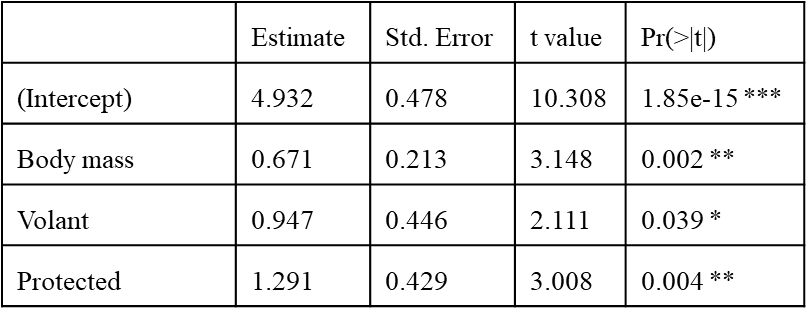


Supplementary table 10a, 10b and 10c. 10a) Model selection output for the best fit model for maximum seed dispersal distance GLM with body mass, volant, protected and HFI as predictors. 10b) AIC scores to determine the best model, with or without an interaction term, for predicting maximum seed dispersal distance GLM with body mass and volant as predictors.10c) Coefficient output table for maximum seed dispersal distance GLM with body mass, volant and an interaction term as predictors.


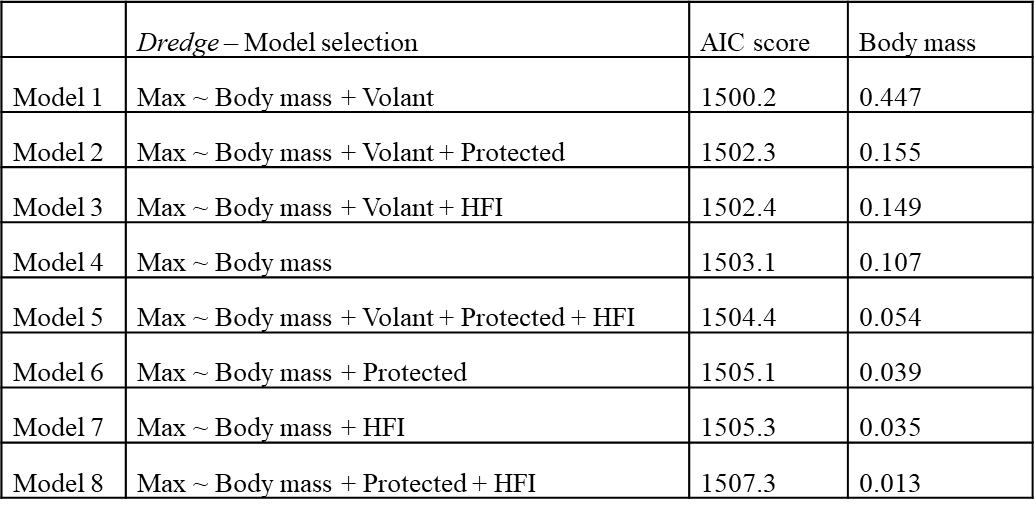


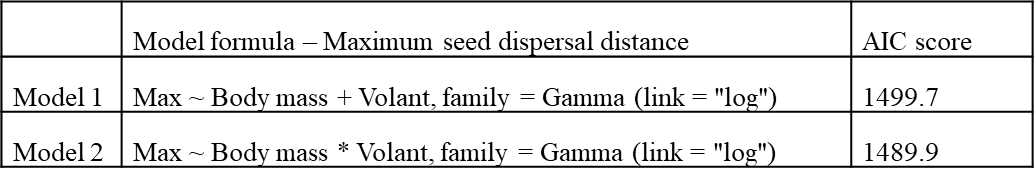


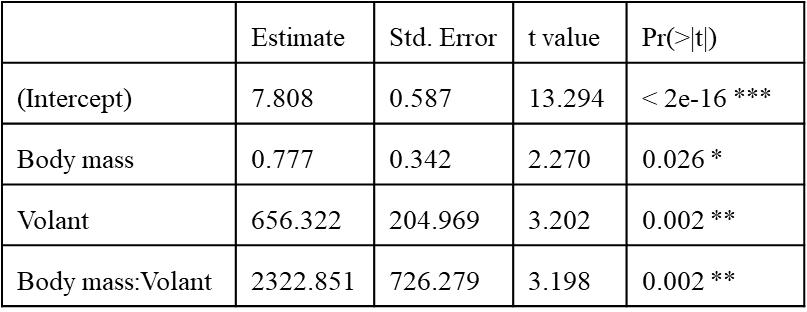


Supplementary table 11a and 11b. 11a) Output for Spearman's rank correlation test between body mass (g) and species average SRT (minutes). 11b) Coefficient output table for the linear model assessing the effect of body mass on the species average SRT.


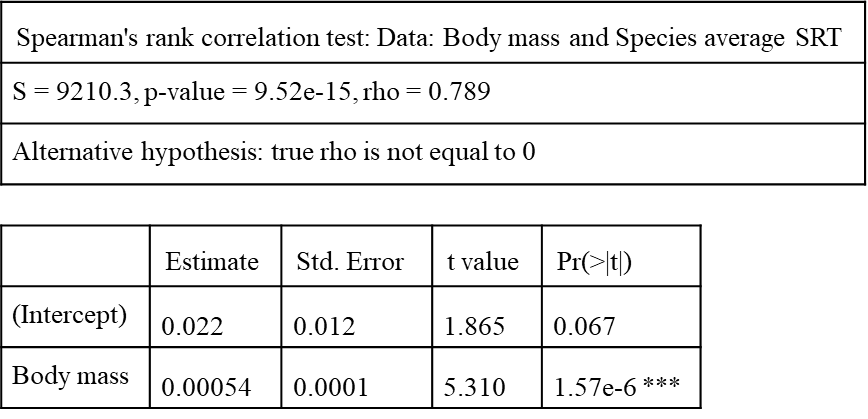


Supplementary table 12. The subset of studies used for each analysis and the reasoning behind the omission of certain studies.


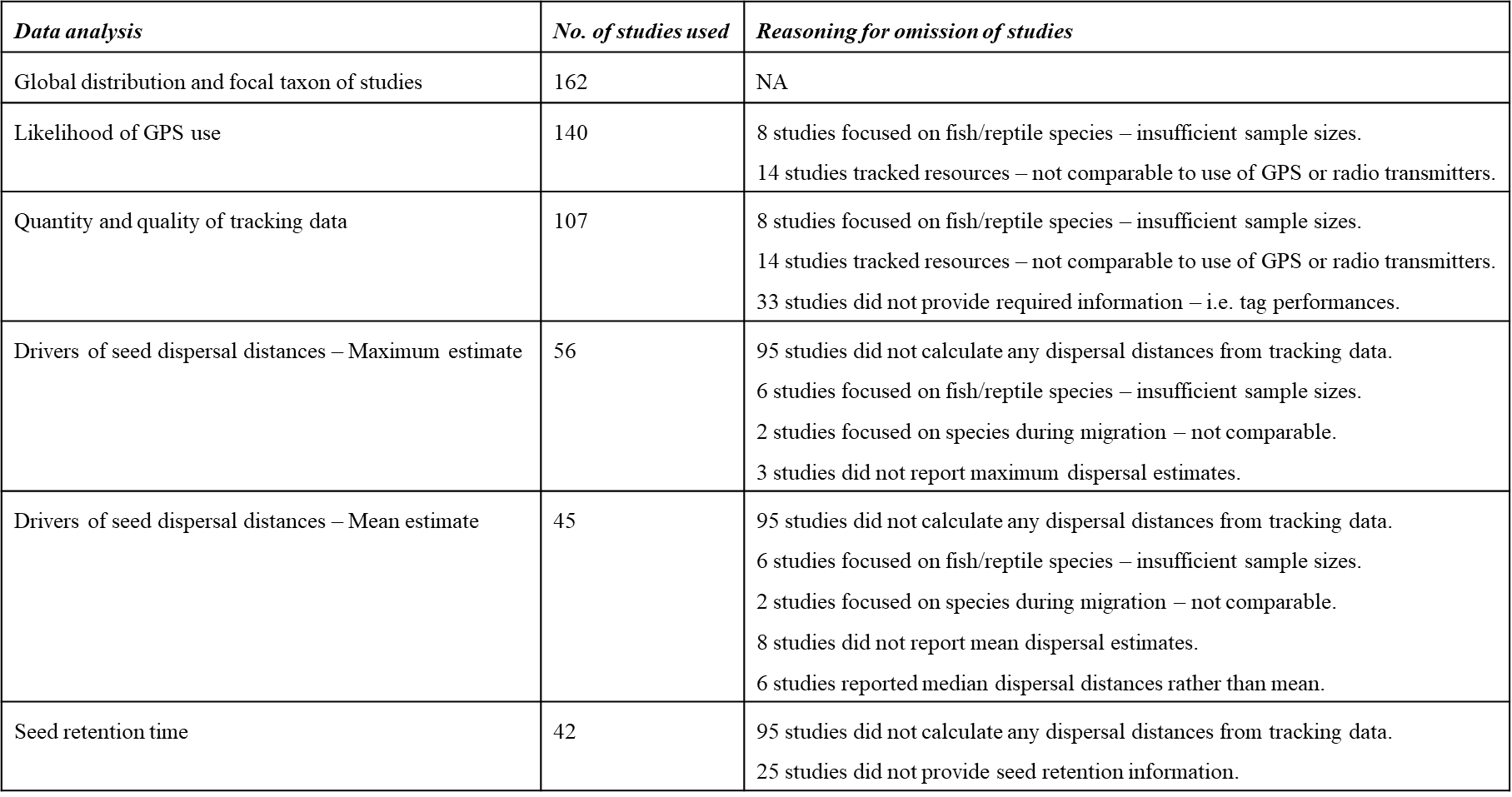


Supplementary table 13. List of 162 frugivore tracking studies included in the review. Information includes Type (if the study only tracked the frugivore or if it also estimated seed dispersal distances), Year of study, Tracking method, Location (the country where the study was carried out in) and Species.

| ***Study***  ***/Reference*** | ***Type*** | ***Year*** | ***Tracking method*** | ***Location*** | ***Species*** |
| --- | --- | --- | --- | --- | --- |
| 1 | Track | 2002 | Radio Tracking | Cameroon | Brown cheeked hornbill |
| 1 | Track | 2002 | Radio Tracking | Cameroon | Black-casqued hornbill |
| 2 | Track | 2014 | GPS | Cameroon | White-thighed hornbill |
| 2 | Track | 2014 | GPS | Cameroon | Black-casqued hornbill |
| 3 | Track | 2008 | GPS | Congo | African bush elephant |
| 3 | Track | 2008 | GPS | Gabon |  |
| 4 | Track | 2011 | Radio Tracking | Madagascar | Madagascan rousette |
| 5 | Track | 2019 | GPS | Mauritius | Mauritian flying fox |
| 6 | Track | 2013 | Radio Tracking | South Africa | Wahlberg's epauletted fruit bat |
| 7 | Track | 2014 | Radio Tracking | South Africa | Wahlberg's epauletted fruit bat |
| 7 | Track | 2014 | Radio Tracking | South Africa | Peter's epauletted fruit bat |
| 8 | Track | 2017 | GPS | South Africa | Trumpeter hornbill |
| 9 | Track | 2016 | GPS | Canary Islands | Elenora falcon |
| 10 | Track | 2019 | Resource tracking | Gabon | African bush elephant |
| 11 | Track | 2012 | Radio tracking | Madagascar | Red-ruffed lemur |
| 12 | Track | 2017 | Radio Tracking | Borneo | Binturong |
| 13 | Track | 2018 | GPS | Mongolia | Bar-headed goose |
| 13 | Track | 2018 | GPS | China |  |
| 13 | Track | 2018 | GPS | India |  |
| 14 | Track | 2016 | Radio Tracking | Cyprus | Egyptian fruit bat |
| 15 | Track | 1998 | Radio Tracking | India | Indian short-nosed fruit bat |
| 16 | Track | 2018 | GPS | Malaysia | Asian elephant |
| 17 | Track | 2015 | GPS | Thailand | Lyle's flying fox |
| 18 | Track | 1994 | Radio Tracking | Thailand | Great hornbill |
| 18 | Track | 1994 | Radio Tracking | Thailand | Brown hornbill |
| 18 | Track | 1994 | Radio Tracking | Thailand | Wreathed hornbill |
| 19 | Track | 2004 | Radio Tracking | Vietnam | Pygmy Loris |
| 20 | Track | 1997 | Resource tracking | Japan | Japanese squirrel |
| 20 | Track | 1997 | Resource tracking | Japan | Field mouse |
| 21 | Track | 2000 | Radio Tracking | Belize | Little yellow-shouldered bat |
| 22 | Track | 1978 | Radio Tracking | Costa Rica | Seba's short-tailed bat |
| 23 | Track | 1986 | Radio Tracking | Costa Rica | Seba's short-tailed bat |
| 24 | Track | 1994 | Radio Tracking | Costa Rica | Resplendent Quetzal |
| 25 | Track | 1997 | Radio Tracking | Costa Rica | *Brycon guatemalensis* |
| 26 | Track | 2004 | Radio Tracking | Costa Rica | Three-wattled bellbird |
| 27 | Track | 2011 | Radio Tracking | Costa Rica | Blue-throated toucanet |
| 28 | Track | 2015 | Radio Tracking | Costa Rica | Thomas's fruit-eating bat |
| 29 | Track | 2007 | Radio Tracking | Costa Rica | Wood thrush |
| 30 | Track | 2007 | Radio Tracking | Costa Rica | Thomas's fruit-eating bat |
| 31 | Track | 2010 | Radio Tracking | Costa Rica | Commissaris's long-tongued bat |
| 32 | Track | 2006 | Radio Tracking | Costa Rica | Silver-throated tanager |
| 32 | Track | 2006 | Radio Tracking | Costa Rica | Orange-billed nightingale-thrush |
| 32 | Track | 2006 | Radio Tracking | Costa Rica | White-throated thrush |
| 33 | Track | 1978 | Radio Tracking | Panama | Jamaican fruit bat |
| 34 | Track | 1991 | Radio Tracking | Panama | Jamaican fruit bat |
| 35 | Track | 2005 | Radio Tracking | Panama | Spiny rat |
| 36 | Track | 2006 | Radio Tracking | Panama | White-throated round-eared bat |
| 37 | Track | 2008 | Radio Tracking | Panama | Agouti |
| 38 | Track | 2016 | GPS | Panama | White-faced capuchin monkey |
| 38 | Track | 2016 | GPS | Panama | Black-handed spider monkey |
| 39 | Track | 2006 | Radio Tracking | Panama | Chestnut short-tailed bat |
| 40 | Track | 2018 | GPS | France | Roe deer |
| 41 | Track | 2012 | Radio tracking | Menorca | Lilford’s wall lizard |
| 42 | Track | 2011 | Radio Tracking | Portugal | Wood mouse |
| 43 | Track | 2013 | Radio Tracking | Sweden | Siberian jay |
| 44 | Track | 2005 | Radio Tracking | USA | Swainson’s thrush |
| 44 | Track | 2005 | Radio Tracking | USA |  |
| 45 | Track | 2018 | GPS | Canada | Grizzly bear |
| 46 | Track | 2005 | Radio Tracking | Dominican Republic | Hispaniolan parrot |
| 47 | Track | 1994 | Radio Tracking | USA | White-crowned pigeon |
| 48 | Track | 2014 | Radio Tracking | USA | Japanese white eye |
| 49 | Track | 1998 | Radio Tracking | Mexico | Lesser long-nosed bat |
| 50 | Track | 2001 | Radio Tracking | Mexico | Keel-billed toucan |
| 51 | Track | 2014 | Radio Tracking | Mexico | Highland yellow-shouldered bat |
| 52 | Track | 1978 | Radio tracking | Mexico | Jamaican fruit bat |
| 53 | Track | 2016 | Radio Tracking | USA | Cedar waxwing |
| 53 | Track | 2016 | Radio Tracking | USA | American robin |
| 54 | Track | 2018 | Radio Tracking | USA | Swainson’s thrush |
| 54 | Track | 2018 | Radio Tracking | USA | Cray catbird |
| 55 | Track | 2005 | Radio tracking | USA | American robin |
| 56 | Track | 1997 | Radio Tracking | Puerto Rico | Red fig-eating bat |
| 57 | Track | 2009 | Radio Tracking | USA | Clarks Nutcracker |
| 58 | Track | 2010 | Radio Tracking | USA | Clarks Nutcracker |
| 59 | Track | 2016 | Radio Tracking | USA | Clarks Nutcracker |
| 60 | Track | 1997 | Radio Tracking | New Zealand | North Island Kaka |
| 61 | Track | 2008 | Radio Tracking | New Zealand | New Zealand pigeon |
| 62 | Track | 2010 | Radio Tracking | New Zealand | North island robin |
| 63 | Track | 1987 | Radio Tracking | Papa New Guinea | Buff-tailed sicklebill |
| 64 | Track | 2002 | Radio Tracking | Papa New Guinea | Lesser bare-backed bat |
| 65 | Track | 2008 | Radio Tracking | Bolivia | Little yellow-shouldered bat |
| 66 | Track | 2010 | Radio Tracking | Bolivia | Red-fronted macaw |
| 67 | Track | 2003 | Radio Tracking | Brazil | Gervais's fruit-eating bat |
| 67 | Track | 2003 | Radio Tracking | Brazil | Striped hair-nosed bat |
| 67 | Track | 2003 | Radio Tracking | Brazil | Silky short-tailed bat |
| 67 | Track | 2003 | Radio Tracking | Brazil | Seba's short-tailed bat |
| 67 | Track | 2003 | Radio Tracking | Brazil | Striped-headed round-eared bat |
| 67 | Track | 2003 | Radio Tracking | Brazil | White-throated round-eared bat |
| 67 | Track | 2003 | Radio Tracking | Brazil | Fringed-lipped bat |
| 67 | Track | 2003 | Radio Tracking | Brazil | Lesser bulldog bat |
| 68 | Track | 2003 | Radio Tracking | Brazil | Agouti |
| 69 | Track | 2008 | Radio Tracking | Brazil | Little yellow-shouldered bat |
| 70 | Track | 2011 | Radio Tracking | Brazil | Black eared opossum |
| 71 | Track | 2013 | Radio Tracking | Brazil | Seba's short-tailed bat |
| 71 | Track | 2013 | Radio Tracking | Brazil | Great fruit-eating bat |
| 72 | Track | 2014 | Radio Tracking | Brazil | Dekeyser's nectar bat |
| 72 | Track | 2014 | Radio Tracking | Brazil | Pallas's long-tongued bat |
| 73 | Track | 2016 | Radio Tracking | Brazil | Pale-breasted thrush |
| 73 | Track | 2016 | Radio Tracking | Brazil | Rufous-bellied thrush |
| 74 | Track | 2009 | Radio Tracking | Brazil | Blue manakin |
| 74 | Track | 2009 | Radio Tracking | Brazil | White-shouldered fire-eye |
| 74 | Track | 2009 | Radio Tracking | Brazil | Rufous-breasted leaftosser |
| 75 | Track | 2013 | Radio tracking | Brazil | White-bearded manakin |
| 76 | Track | 2013 | Radio Tracking | Chile | Austral thrush |
| 77 | Track | 2016 | Radio Tracking | Chile | Monito del monte |
| 78 | Track | 2017 | GPS | Colombia | South American tapir |
| 79 | Track | 2018 | GPS | Colombia | White-footed tamarin |
| 80 | Track | 2007 | Radio Tracking | Ecuador | Chestnut short-tailed bat |
| 80 | Track | 2007 | Radio Tracking | Ecuador | Seba's short-tailed bat |
| 81 | Track | 1991 | Radio tracking | French Guiana | Seba's short-tailed bat |
| 82 | Track | 2007 | Radio tracking | French Guiana | Dwarf little fruit bat |
| 83 | Track | 1999 | Radio Tracking | French Guiana | Bare-tailed woolly opossum |
| 84 | Track | 2007 | Radio Tracking | French Guiana | Dwarf little fruit bat |
| 85 | Track | 1992 | Radio Tracking | French Guiana | White-crowned manakin |
| 85 | Track | 1992 | Radio Tracking | French Guiana | Golden-headed manakin |
| 85 | Track | 1992 | Radio Tracking | French Guiana | White-fronted manakin |
| 85 | Track | 1992 | Radio Tracking | French Guiana | White-bearded manakin |
| 85 | Track | 1992 | Radio Tracking | French Guiana | Thrush-like manakin |
| 86 | Track | 2018 | Radio Tracking | Peru & Ecuador | Shining sunbeam hummingbird |
| 87 | Track | 2009 | GPS | Venezuela | Oilbird |
| 88 | Track | 2021 | Radio tracking | Brazil | Little yellow-shouldered bat |
| 89 | Track | 2020 | GPS | Brazil | White-lipped peccary |
| 90 | Track | 2020 | GPS | Tanzania | Straw-coloured fruit bat |
| 91 | Track | 2018 | GPS | Gabon | African forest elephant |
| 92 | Track | 2022 | GPS | Panama | White-faced capuchin monkey |
| 92 | Track | 2022 | GPS | Panama | Kinkajou |
| 92 | Track | 2022 | GPS | Panama | Coati |
| 92 | Track | 2022 | GPS | Panama | Black-handed spider monkey |
| 93 | Track | 2021 | GPS | Myanmar | Indian flying fox |
| 94 | Track | 2020 | GPS | Australia | Little red flying fox |
| 94 | Track | 2020 | GPS | Australia | Black flying fox |
| 94 | Track | 2020 | GPS | Australia | Grey-headed flying fox |
| 95 | Track | 2020 | Radio tracking | Costa Rica | Pygmy fruit-eating bat |
| 96 | Seed | 2000 | Radio tracking | Cameroon | White-thighed hornbill |
| 96 | Seed | 2000 | Radio tracking | Cameroon | Black-casqued hornbill |
| 97 | Seed | 2009 | GPS | Congo | African forest elephant |
| 98 | Seed | 2016 | GPS | Ghana | Straw-coloured fruit bat |
| 99 | Seed | 2009 | Radio tracking | Kenya | Stripe-cheeked greenbul |
| 99 | Seed | 2009 | Radio tracking | Kenya | Taita thrush |
| 99 | Seed | 2009 | Radio tracking | Kenya | Hartlaub's turaco |
| 100 | Seed | 2018 | Radio tracking | Madagascar | Common brown lemur |
| 101 | Seed | 2017 | GPS | Madagascar | Madagascan flying fox |
| 102 | Seed | 2011 | GPS | South Africa | Trumpeter hornbill |
| 103 | Seed | 2014 | GPS | South Africa | Trumpeter hornbill |
| 104 | Seed | 2017 | GPS | South Africa | African bush elephant |
| 105 | Seed | 2016 | GPS | South Africa | Red-billed teal |
| 105 | Seed | 2016 | GPS | South Africa | Egyptian geese |
| 105 | Seed | 2016 | GPS | South Africa |  |
| 106 | Seed | 2009 | Radio tracking | Borneo | Common palm civet |
| 107 | Seed | 2007 | Radio tracking | Hong Kong | Red-whiskered Bulbul |
| 107 | Seed | 2007 | Radio tracking | Hong Kong | Light-vented Bulbul |
| 107 | Seed | 2007 | Radio tracking | Hong Kong | Chinese Hwamei |
| 108 | Seed | 2019 | GPS | India | Wreathed Hornbill |
| 108 | Seed | 2019 | GPS | India | Great Hornbill |
| 109 | Seed | 2015 | GPS | India | Asian elephant |
| 110 | Seed | 2007 | Radio tracking | Israel | Yellow-vented Bulbul |
| 110 | Seed | 2007 | Radio tracking | Israel | Tristram's Grackle |
| 111 | Seed | 2010 | GPS | Israel | Egyptian fruit bat |
| 112 | Seed | 2009 | Radio tracking | Japan | Orii's flying fox |
| 113 | Seed | 2016 | GPS | Japan | Raccoon Dog |
| 114 | Seed | 2010 | GPS | Japan | Asiatic black bear |
| 115 | Seed | 1996 | Resource tracking | Japan | Field mouse |
| 116 | Seed | 2008 | Resource tracking | Japan | Field mouse |
| 116 | Seed | 2008 | Resource tracking | Japan | Japanese squirrel |
| 117 | Seed | 2011 | GPS | Malaysia | Malayan tapir |
| 117 | Seed | 2011 | Radio tracking | Malaysia | Malayan tapir |
| 118 | Seed | 2008 | GPS | Sri Lanka | Asian elephant |
| 118 | Seed | 2008 | GPS | Myanmar |  |
| 119 | Seed | 1988 | Radio tracking | Costa Rica | Black-faced solitaire |
| 119 | Seed | 1988 | Radio tracking | Costa Rica | Black and yellow phainoptila |
| 119 | Seed | 1988 | Radio tracking | Costa Rica | Prong-billed barbet |
| 120 | Seed | 2000 | Radio tracking | Costa Rica | Ochre-bellied flycatcher |
| 121 | Seed | 2015 | Radio tracking | Costa Rica | Blue-crowned manakin |
| 121 | Seed | 2015 | Radio tracking | Costa Rica | White-ruffed manakin |
| 122 | Seed | 2011 | GPS | Panama | Keel-billed toucan |
| 122 | Seed | 2011 | GPS | Panama | Chesnut Mandibled Toucan |
| 123 | Seed | 2012 | Resource tracking | Panama | Agouti |
| 124 | Seed | 2018 | Resource tracking | Austria | Common pine vole |
| 124 | Seed | 2018 | Resource tracking | Austria | Bank vole |
| 124 | Seed | 2018 | Resource tracking | Austria | Yellow-necked mouse |
| 124 | Seed | 2018 | Resource tracking | Austria | Common dormouse |
| 124 | Seed | 2018 | Resource tracking | Austria | Wood mouse |
| 124 | Seed | 2018 | Resource tracking | Austria | Edible dormouse |
| 125 | Seed | 2016 | GPS | France | Roe deer |
| 125 | Seed | 2016 | GPS | France | Wild Boar |
| 125 | Seed | 2016 | GPS | France | Red Deer |
| 126 | Seed | 2012 | Radio tracking | Germany | Blackbird |
| 127 | Seed | 2015 | GPS | Netherlands | Mallard |
| 128 | Seed | 2019 | GPS | Germany | Mallard |
| 129 | Seed | 2018 | Resource tracking | Poland | European Jay |
| 130 | Seed | 2012 | Radio tracking | Spain | Eyed lizard |
| 131 | Seed | 2007 | Resource tracking | Spain | European Jay |
| 132 | Seed | 2017 | Resource tracking | Spain | European magpie |
| 133 | Seed | 2015 | Resource tracking | Spain | European Jay |
| 134 | Seed | 2007 | Radio tracking | Spain | Liford's wall lizard |
| 135 | Seed | 2013 | Radio tracking | USA | Japanese white eye |
| 135 | Seed | 2013 | Radio tracking | USA | Oma'o |
| 136 | Seed | 2018 | Resource tracking | USA | White-footed mouse |
| 136 | Seed | 2018 | Resource tracking | USA | Chipmunk |
| 136 | Seed | 2018 | Resource tracking | USA | Grey squirrel |
| 137 | Seed | 2016 | Radio tracking | USA | Wood thrush |
| 138 | Seed | 2011 | Radio tracking | Australia | Spiny-cheeked honeyeater |
| 139 | Seed | 2020 | GPS | Australia | Emu |
| 140 | Seed | 2007 | Radio tracking | Australia | Mistletoe bird |
| 141 | Seed | 2008 | Radio tracking | Australia | Painted honeyeater |
| 142 | Seed | 1991 | Radio tracking | Australia | Tooth-billed bowerbird |
| 143 | Seed | 2005 | Radio tracking | Australia | Cassowaries |
| 144 | Seed | 2018 | Radio tracking | New Caledonia | Red-vented bulbul |
| 145 | Seed | 2012 | Radio tracking | New Zealand | Kereru |
| 146 | Seed | 2003 | Resource tracking | Papa New Guinea | Cassowaries |
| 147 | Seed | 2019 | Radio tracking | Saipan | Bridled white eye |
| 147 | Seed | 2019 | Radio tracking | Saipan | Golden white eye |
| 147 | Seed | 2019 | Radio tracking | Saipan | Micronesian starling |
| 147 | Seed | 2019 | Radio tracking | Saipan | Maria Fruit dove |
| 147 | Seed | 2019 | Radio tracking | Saipan | White-throated ground dove |
| 148 | Seed | 2009 | Radio tracking | Brazil | Yellow-footed tortoise |
| 149 | Seed | 2011 | Radio tracking | Brazil | White-crowned manakin |
| 149 | Seed | 2011 | Radio tracking | Brazil | White-fronted manakin |
| 149 | Seed | 2011 | Radio tracking | Brazil | Golden-headed manakin |
| 149 | Seed | 2011 | Radio tracking | Brazil | Thrush-like manakin |
| 149 | Seed | 2011 | Radio tracking | Brazil | White-necked thrush |
| 150 | Seed | 2019 | Radio tracking | Chile | Monito del monte |
| 151 | Seed | 2012 | Radio tracking | Ecuador | Umbrellabird |
| 152 | Seed | 2016 | Radio tracking | Ecuador | White-crowned manakin |
| 153 | Seed | 2011 | Radio tracking | Ecuador | Many-banded aracari |
| 153 | Seed | 2011 | Radio tracking | Ecuador | Channel-billed toucan |
| 153 | Seed | 2011 | Radio tracking | Ecuador | White-throated toucan |
| 154 | Seed | 1993 | Radio tracking | French Guiana | Kinkajou |
| 155 | Seed | 2008 | Radio tracking | Peru | Yellow-footed tortoise |
| 156 | Seed | 2011 | Radio tracking | Peru | Black pacu |
| 157 | Seed | 2022 | Resource tracking | Poland | European Jay |
| 158 | Seed | 2021 | GPS | Gabon | African forest elephant |
| 159 | Seed | 2021 | GPS | Colombia | Oilbird |
| 160 | Seed | 2022 | GPS | Spain | Yellow-legged gull |
| 161 | Seed | 2020 | Radio tracking | Indonesia | Southeast Asian box turtle |
| 162 | Seed | 2021 | Resource tracking | Spain | European magpie |

Reference list for all 162 studies used in this review – Number corresponds to study number in Supplementary Table 13.

1. Holbrook, K. M., Smith, T. B., & Hardesty, B. D. (2002). Implications of long‐distance movements of frugivorous rain forest hornbills. *Ecography*, 25(6), 745-749.
2. Chasar, A., Harrigan, R. J., Holbrook, K. M., Dietsch, T. V., Fuller, T. L., Wikelski, M., & Smith, T. B. (2014). Spatial and temporal patterns of frugivorous hornbill movements in Central Africa and their implications for rain forest conservation. *Biotropica*, 46(6), 763-770.
3. Blake, S., Deem, S. L., Strindberg, S., Maisels, F., Momont, L., Isia, I. B., & Kock, M. D. (2008). Roadless wilderness area determines forest elephant movements in the Congo Basin. *PloS one*, 3(10), e3546.
4. Andrianaivoarivelo, R. A., Ramilijaona, O. R., Racey, P. A., Razafindrakoto, N., & Jenkins, R. K. (2011). Feeding ecology, habitat use and reproduction of *Rousettus madagascariensis Grandidier*, 1928 (*Chiroptera*: *Pteropodidae*) in eastern Madagascar.
5. Oleksy, R. Z., Ayady, C. L., Tatayah, V., Jones, C., Howey, P. W., Froidevaux, J. S., & Jones, G. (2019). The movement ecology of the Mauritian flying fox (*Pteropus niger*): a long-term study using solar-powered GSM/GPS tags. *Movement Ecology*, 7(1), 1-12.
6. Rollinson, D. P., Coleman, J. C., & Downs, C. T. (2013). Seasonal differences in foraging dynamics, habitat use and home range size of Wahlberg's epauletted fruit bat in an urban environment. *African Zoology*, 48(2), 340-350.
7. Bonaccorso, F. J., Winkelmann, J. R., Todd, C. M., & Miles, A. C. (2014). Foraging movements of epauletted fruit bats (*Pteropodidae*) in relation to the distribution of sycamore figs (*Moraceae*) in Kruger National Park, South Africa. *Acta Chiropterologica*, 16(1), 41-52.
8. Chibesa, M., Taylor, B., Ramesh, T., & Downs, C. T. (2017). Home range and habitat use of Trumpeter Hornbills *Bycanistes bucinator* in an urban–forest mosaic, Eshowe, South Africa. *Ostrich*, 88(3), 225-233.
9. Viana, D. S., Gangoso, L., Bouten, W., & Figuerola, J. (2016). Overseas seed dispersal by migratory birds. *Proceedings of the Royal Society B: Biological Sciences*, 283(1822), 20152406.
10. Beirne, C., Nuñez, C. L., Baldino, M., Kim, S., Knorr, J., Minich, T., & Poulsen, J. R. (2019). Estimation of gut passage time of wild, free roaming forest elephants. *Wildlife Biology*, 2019(1), 1-7.
11. Razafindratsima, O. H., & Martinez, B. T. (2012). Seed dispersal by red-ruffed lemurs: seed size, viablity, and beneficial effect on seedling growth. *Ecotropica*, 18(1), 15-26.
12. Nakabayashi, M., Ahmad, A. H., & Kohshima, S. (2017). Fruit selection of a binturong (*Arctictis binturong*) by focal animal sampling in Sabah, Malaysian Borneo. *Mammalia*, 81(1), 107-110.
13. van Toor, M. L., Kranstauber, B., Newman, S. H., Prosser, D. J., Takekawa, J. Y., Technitis, G., & Safi, K. (2018). Integrating animal movement with habitat suitability for estimating dynamic migratory connectivity. *Landscape Ecology,* 33, 879-893.
14. Lučan, R. K., Bartonička, T., Jedlička, P., Řeřucha, Š., Šálek, M., Čížek, M., & Horáček, I. (2016). Spatial activity and feeding ecology of the endangered northern population of the Egyptian fruit bat (*Rousettus aegyptiacus*). *Journal of Mammalogy*, 97(3), 815-822.
15. Gopukumar Nair, N., Elangovan, V., & Subbaraj, R. (1998). Influence of moonlight on the foraging behaviour of the Indian Short-nosed fruit bat, *cynopterus sphinx*: radio-telemetry studies. *Current Science*, 74(8), 688-689.
16. Wadey, J., Beyer, H. L., Saaban, S., Othman, N., Leimgruber, P., & Campos-Arceiz, A. (2018). Why did the elephant cross the road? The complex response of wild elephants to a major road in Peninsular Malaysia. *Biological Conservation*, 218, 91-98.
17. Weber, N., Duengkae, P., Fahr, J., Dechmann, D. K., Phengsakul, P., Khumbucha, W., & Newman, S. (2015). High‐resolution GPS tracking of Lyle's flying fox between temples and orchards in central Thailand. *The Journal of Wildlife Management*, 79(6), 957-968.
18. Poonswad, P., & Tsuji, A. (1994). Ranges of males of the great hornbill *Buceros bicornis*, brown hornbill *Ptilolaemus tickelli* and wreathed hornbill *Rhyticeros undulatus* in Khao Yai National Park, Thailand. *Ibis*, 136(1), 79-86.
19. Streicher, U. (2009). Diet and feeding behaviour of pygmy lorises (*Nycticebus pygmaeus*) in Vietnam. *Vietnamese Journal of Primatology*, 3, 37-44.
20. Tamura, N. (1997). Japanese squirrels as a seed disperser of walnuts. *Primate Research*, 13(2), 129-135.
21. Fenton, M. B., Vonhof, M. J., Bouchard, S., Gill, S. A., Johnston, D. S., Reid, F. A., & Wagner, R. (2000). Roosts Used by *Sturnira lilium (Chiroptera: Phyllostomidae)* in Belize. *Biotropica*, 32(4a), 729-733.
22. Heithaus, E. R., & Fleming, T. H. (1978). Foraging movements of a frugivorous bat, *Carollia perspicillata (Phyllostomatidae)*. *Ecological Monographs*, 48(2), 127-143.
23. Fleming, T. H., & Heithaus, E. R. (1986). Seasonal foraging behavior of the frugivorous bat *Carollia perspicillata.* *Journal of Mammalogy*, 67(4), 660-671.
24. Powell, G. V., & Bjork, R. D. (1994). Implications of altitudinal migration for conservation strategies to protect tropical biodiversity: a case study of the Resplendent Quetzal *Pharomacrus mocinno* at Monteverde, Costa Rica. *Bird Conservation International*, 4(2-3), 161-174.
25. Horn, M. H. (1997). Evidence for dispersal of fig seeds by the fruit-eating characid fish *Brycon guatemalensis* Regan in a Costa Rican tropical rain forest. *Oecologia*, 109, 259-264.
26. Powell, G. V., & Bjork, R. D. (2004). Habitat linkages and the conservation of tropical biodiversity as indicated by seasonal migrations of Three‐wattled Bellbirds. *Conservation Biology*, 18(2), 500-509.
27. Peters, V. E., & Nibbelink, N. (2011). The value of fruit security for the conservation of a neotropical frugivore in human-dominated landscapes. *Biodiversity and Conservation*, 20, 2041-2055.
28. Ripperger, S. P., Kalko, E. K., Rodríguez-Herrera, B., Mayer, F., & Tschapka, M. (2015). Frugivorous bats maintain functional habitat connectivity in agricultural landscapes but rely strongly on natural forest fragments. *PloS one*, 10(4), e0120535.
29. Roberts, D. L. (2007). Effects of tropical forest fragmentation on ecology and conservation of migrant and resident birds in lowland Costa Rica.
30. Chaverri, G., Quirós, O. E., & Kunz, T. H. (2007). Ecological correlates of range size in the tent-making bat *Artibeus watsoni*. *Journal of mammalogy*, 88(2), 477-486.
31. Rothenwöhrer, C., Becker, N. I., & Tschapka, M. (2011). Resource landscape and spatio‐temporal activity patterns of a plant‐visiting bat in a Costa Rican lowland rainforest. *Journal of Zoology*, 283(2), 108-116.
32. Şekercioğlu, C. H., Loarie, S. R., Oviedo Brenes, F., Ehrlich, P. R., & Daily, G. C. (2007). Persistence of forest birds in the Costa Rican agricultural countryside. *Conservation Biology*, 21(2), 482-494.
33. Morrison, D. W. (1978). Foraging ecology and energetics of the frugivorous bat *Artibeus jamaicensis. Ecology,* 59(4), 716-723.
34. Handley Jr, C. O., Wilson, D. E., & Gardner, A. L. (1991). Demography and natural history of the common fruit bat, *Artibeus jamaicensis*, on Barro Colorado Island, Panamá.
35. Endries, M. J., & Adler, G. H. (2005). Spacing patterns of a tropical forest rodent, the spiny rat *(Proechimys semispinosus)*, in Panama*. Journal of Zoology*, 265(2), 147-155.
36. Lang, A. B., Kalko, E. K., Römer, H., Bockholdt, C., & Dechmann, D. K. (2006). Activity levels of bats and katydids in relation to the lunar cycle. *Oecologia*, 146, 659-666.
37. Aliaga-Rossel, E., Kays, R. W., & Fragoso, J. M. (2008). Home-range use by the central American agouti (*Dasyprocta punctata*) on Barro Colorado Island, Panama*. Journal of Tropical Ecology,* 24(4), 367-374.
38. McLean, K. A., Trainor, A. M., Asner, G. P., Crofoot, M. C., Hopkins, M. E., Campbell, C. J., & Jansen, P. A. (2016). Movement patterns of three arboreal primates in a Neotropical moist forest explained by LiDAR-estimated canopy structure*. Landscape Ecology*, 31, 1849-1862.
39. Thies, W., Kalko, E. K., & Schnitzler, H. U. (2006). Influence of environment and resource availability on activity patterns of *Carollia castanea (Phyllostomidae*) in Panama. *Journal of Mammalogy*, 87(2), 331-338.
40. Martin, J., Vourc’h, G., Bonnot, N., Cargnelutti, B., Chaval, Y., Lourtet, B., & Morellet, N. (2018). Temporal shifts in landscape connectivity for an ecosystem engineer, the roe deer, across a multiple-use landscape*. Landscape ecology*, 33, 937-954.
41. Rodríguez‐Pérez, J., Wiegand, T., & Santamaria, L. (2012). Frugivore behaviour determines plant distribution: a spatially‐explicit analysis of a plant‐disperser interaction. *Ecography*, 35(2), 113-123.
42. Rosalino, L. M., Ferreira, D., Leitão, I., & Santos-Reis, M. (2011). Selection of nest sites by wood mice *Apodemus sylvaticus* in a Mediterranean agro-forest landscape. *Ecological research,* 26, 445-452.
43. Griesser, M., Halvarsson, P., Sahlman, T., & Ekman, J. (2014). What are the strengths and limitations of direct and indirect assessment of dispersal? Insights from a long-term field study in a group-living bird species. *Behavioral Ecology and Sociobiology*, 68, 485-497.
44. White, J. D., Gardali, T., Thompson III, F. R., & Faaborg, J. (2005). Resource selection by juvenile Swainson's Thrushes during the postfledging period. *The Condor*, 107(2), 388-401.
45. Denny, C. K., Stenhouse, G. B., & Nielsen, S. E. (2018). Scales of selection and perception: landscape heterogeneity of an important food resource influences habitat use by a large omnivore. *Wildlife Biology,* 2018(1), 1-10.
46. White, T. H., Collazo, J. A., Vilella, F. J., & Guerrero, S. A. (2005). Effects of Hurricane Georges on habitat use by captive-reared Hispaniolan Parrots (*Amazona ventralis*) released in the Dominican Republic.
47. Strong, A. M., & Bancroft, G. T. (1994). Post-fledging Dispersal of White‐crowned Pigeons: Implications for Conservation of Deciduous Seasonal Forests in the Florida Keys. *Conservation Biology,* 8(3), 770-779.
48. Aslan, A., Hart, P., Wu, J., & Aslan, C. E. (2014). Evaluating the qualitative effectiveness of a novel pollinator: a case study of two endemic Hawaiian plants. *Biotropica*, 46(6), 732-739.
49. Horner, M. A., Fleming, T. H., & Sahey, C. T. (1998). Foraging behaviour and energetics of a nectar-feeding bat, *Leptonycteris curasoae (Chiroptera: Phyllostomidae*). *Journal of Zoology*, 244(4), 575-586.
50. Graham, C. H. (2001). Factors influencing movement patterns of keel‐billed toucans in a fragmented tropical landscape in southern Mexico. *Conservation Biology*, 15(6), 1789-1798.
51. Cortés‐Delgado, N., & Sosa, V. J. (2014). Do bats roost and forage in shade coffee plantations? A perspective from the frugivorous bat *Sturnira hondurensis. Biotropica*, 46(5), 624-632.
52. Morrison, D. W. (1978). Influence of habitat on the foraging distances of the fruit bat, *Artibeus jamaicensis. Journal of Mammalogy*, 59(3), 622-624.
53. Eaton, R. A., Lindell, C. A., Homan, H. J., Linz, G. M., & Maurer, B. A. (2016). American Robins (*Turdus migratorius*) and Cedar Waxwings (*Bombycilla cedrorum*) vary in use of cultivated cherry orchards. *The Wilson Journal of Ornithology*, 128(1), 97-107.
54. Oguchi, Y., Pohlen, Z., Smith, R. J., & Owen, J. C. (2018). Exotic-and native-dominated shrubland habitat use by fall migrating Swainson's Thrushes and Gray Catbirds in Michigan, USA. *The Condor: Ornithological Applications*, 120(1), 81-93.
55. Bartuszevige, A. M., & Gorchov, D. L. (2006). Avian seed dispersal of an invasive shrub. *Biological Invasions*, 8, 1013-1022.
56. Gannon, M. R., & Willig, M. R. (1997). The effect of lunar illumination on movement and activity of the red fig-eating bat *(Stenoderma rufum). Biotropica*, 525-529.
57. Lorenz, T. J., & Sullivan, K. A. (2009). Seasonal differences in space use by Clark's Nutcrackers in the Cascade Range. *The Condor*, 111(2), 326-340.
58. Lorenz, T. J., & Sullivan, K. A. (2010). Comparison of survey methods for monitoring Clark's Nutcrackers and predicting dispersal of whitebark pine seeds. *Journal of Field Ornithology*, 81(4), 430-441.
59. Schaming, T. D. (2016). Clark’s nutcracker breeding season space use and foraging behavior. *PLoS One,* 11(2), e0149116.
60. Moorhouse, R. J. (1997). The diet of the North Island kaka (*Nestor meridionalis septentrionalis*) on Kapiti Island. *New Zealand Journal of Ecology*, 141-152.
61. Wotton, D. M., Clout, M. N., & Kelly, D. (2008). Seed retention times in the New Zealand pigeon (*Hemiphaga novaezeelandiae*). *New Zealand Journal of Ecology*, 1-6.
62. Richard, Y., & Armstrong, D. P. (2010). Cost distance modelling of landscape connectivity and gap‐crossing ability using radio‐tracking data. *Journal of Applied Ecology*, 47(3), 603-610.
63. Beehler, B. M. (1987). Ecology and behavior of the Buff-tailed Sicklebill (*Paradisaeidae: Epimachus albertisi). The Auk*, 104(1), 48-55.
64. Bonaccorso, F. J., Winkelmann, J. R., Dumont, E. R., & Thibault, K. (2002). Home range of *Dobsonia minor (Pteropodidae*): a solitary, foliage–roosting fruit bat in Papua New Guinea. *Biotropica*, 34(1), 127-135.
65. Loayza, A. P., & Loiselle, B. A. (2008). Preliminary information on the home range and movement patterns of *Sturnira lilium (Phyllostomidae*) in a naturally fragmented landscape in Bolivia. *Biotropica*, 40(5), 630-635.
66. Meyer, C. (2010). Spatial ecology and conservation of the endemic and endangered Red-fronted Macaw (*Ara rubrogenys*) in the Bolivian Andes. Göttingen, Germany: Georg-August University.
67. Bernard, E., & Fenton, M. B. (2003). Bat mobility and roosts in a fragmented landscape in central Amazonia, Brazil. *Biotropica*, 35(2), 262-277.
68. Silvius, K. M., & Fragoso, J. M. V. (2003). Red‐rumped Agouti (*Dasyprocta leporina*) Home range use in an Amazonian forest: implications for the aggregated distribution of forest trees. *Biotropica*, 35(1), 74-83.
69. Mello, M. A. R., Kalko, E. K. V., & Silva, W. R. (2008). Movements of the bat *Sturnira lilium* and its role as a seed disperser of Solanaceae in the Brazilian Atlantic Forest*. Journal of Tropical Ecology*, 24(2), 225-228.
70. Cerboncini, R. A. S., Passamani, M., & Braga, T. V. (2011). Use of space by the black-eared opossum *Didelphis aurita* in a rural area in southeastern Brazil.
71. Trevelin, L. C., Silveira, M., Port-Carvalho, M., Homem, D. H., & Cruz-Neto, A. P. (2013). Use of space by frugivorous bats *(Chiroptera: Phyllostomidae)* in a restored Atlantic forest fragment in Brazil*. Forest Ecology and Management*, 291, 136-143.
72. Aguiar, L., Bernard, E., & Machado, R. B. (2014). Habitat use and movements of *Glossophaga soricina* and *Lonchophylla dekeyseri (Chiroptera: Phyllostomidae*) in a Neotropical savannah. *Zoologia*, 31, 223-229.
73. Da Silveira, N. S., Niebuhr, B. B. S., Muylaert, R. D. L., Ribeiro, M. C., & Pizo, M. A. (2016). Effects of land cover on the movement of frugivorous birds in a heterogeneous landscape*. PloS one*, 11(6), e0156688.
74. Hansbauer, M. M., Storch, I., Knauer, F., Pilz, S., Küchenhoff, H., Végvári, Z., & Metzger, J. P. (2010). Landscape perception by forest understory birds in the Atlantic Rainforest: black-and-white versus shades of grey. *Landscape ecology,* 25, 407-417.
75. Cestari, C., & Pizo, M. A. (2013). Seed dispersal by the lek-forming white-bearded manakin *(Manacus manacus, Pipridae)* in the Brazilian Atlantic forest. *Journal of Tropical Ecology*, 29(5), 381-389.
76. Vergara, P. M., Pérez-Hernández, C. G., Hahn, I. J., & Jiménez, J. E. (2013). Matrix composition and corridor function for austral thrushes in a fragmented temperate forest. *Landscape Ecology*, 28, 121-133.
77. Salazar, D. A., & Fonturbel, F. E. (2016). Beyond habitat structure: landscape heterogeneity explains the monito del monte (*Dromiciops gliroides*) occurrence and behavior at habitats dominated by exotic trees. *Integrative Zoology*, 11(5), 413-421.
78. González, T. M., González-Trujillo, J. D., Palmer, J. R., Pino, J., & Armenteras, D. (2017). Movement behavior of a tropical mammal: The case of *Tapirus terrestris*. *Ecological Modelling*, 360, 223-229.
79. Sanchez-Giraldo, C., & Daza, J. M. (2019). Getting better temporal and spatial ecology data for threatened species: using lightweight GPS devices for small primate monitoring in the northern Andes of Colombia. *Primates*, 60(1), 93-102.
80. Bonaccorso, F. J., Winkelmann, J. R., Shin, D., Agrawal, C. I., Aslami, N., Bonney, C., ... & Kunz, T. H. (2007). Evidence for exploitative competition: comparative foraging behavior and roosting ecology of short‐tailed fruit bats (*Phyllostomidae). Biotropica*, 39(2), 249-256.
81. Charles-Dominique, P. (1991). Feeding strategy and activity budget of the frugivorous bat *Carollia perspicillata (Chiroptera: Phyllostomidae*) in French Guiana. *Journal of Tropical Ecology*, 7(2), 243-256.
82. Henry, M., & Kalko, E. K. (2007). Foraging strategy and breeding constraints of *Rhinophylla pumilio (Phyllostomidae)* in the Amazon lowlands*. Journal of Mammalogy*, 88(1), 81-93.
83. Julien-Laferriere, D. (1999). Foraging strategies and food partitioning in the neotropical frugivorous mammals *Caluromys philander* and *Potos flavus*. *Journal of Zoology*, 247(1), 71-80.
84. Henry, M., Pons, J. M., & Cosson, J. F. (2007). Foraging behaviour of a frugivorous bat helps bridge landscape connectivity and ecological processes in a fragmented rainforest. *Journal of Animal Ecology*, 76(4), 801-813.
85. Thery, M. (1992). The evolution of leks through female choice: differential clustering and space utilization in six sympatric manakins. *Behavioral Ecology and Sociobiolog*y, 30, 227-237.
86. Hazlehurst, J. A., & Karubian, J. O. (2018). Impacts of nectar robbing on the foraging ecology of a territorial hummingbird. *Behavioural Processes*, 149, 27-34.
87. Holland, R. A., Wikelski, M., Kümmeth, F., & Bosque, C. (2009). The secret life of oilbirds: new insights into the movement ecology of a unique avian frugivore. *PLoS One*, 4(12), e8264.
88. Kerches-Rogeri, P., Ramos, D. L., Siren, J., de Oliveira Teles, B., Alves, R. S. C., Priante, C. F., & Ovaskainen, O. (2021). Movement syndromes of a Neotropical frugivorous bat inhabiting heterogeneous landscapes in Brazil. *Movement Ecology*, 9, 1-12.
89. Jorge, M. L. S., Bradham, J. L., Keuroghlian, A., Oshima, J. E. F., & Ribeiro, M. C. (2021). Permeability of Neotropical agricultural lands to a key native ungulate - Are well‐connected forests important? *Biotropica*, 53(1), 201-212.
90. Randhawa, N., Bird, B. H., VanWormer, E., Sijali, Z., Kilonzo, C., Msigwa, A., & Mazet, J. A. (2020). Fruit bats in flight: a look into the movements of the ecologically important *Eidolon helvum* in Tanzania. *One health outlook*, 2(1), 1-14.
91. Mills, E. (2017). Forest elephant movements and habitat use in a tropical forest-savanna mosaic in Gabon.
92. Havmøller, L. W., Loftus, J. C., Havmøller, R. W., Alavi, S. E., Caillaud, D., Grote, M. N., & Crofoot, M. C. (2021). Arboreal monkeys facilitate foraging of terrestrial frugivores. *Biotropica*, 53(6), 1685-1697.
93. McEvoy, J. F., Kishbaugh, J. C., Valitutto, M. T., Aung, O., Tun, K. Y. N., Win, Y. T., & Murray, S. (2021). Movements of Indian flying fox in Myanmar as a guide to human-bat interface sites. *EcoHealth*, 18(2), 204-216.
94. Welbergen, J. A., Meade, J., Field, H. E., Edson, D., McMichael, L., Shoo, L. P., & Martin, J. M. (2020). Extreme mobility of the world’s largest flying mammals creates key challenges for management and conservation. *BMC biology*, 18(1), 1-13.
95. Villalobos-Chaves, D., Melo, F. P., & Rodríguez-Herrera, B. (2020). Dispersal patterns of large-seeded plants and the foraging behaviour of a frugivorous bat. *Journal of Tropical Ecology*, 36(3), 94-100.
96. Holbrook, K. M., & Smith, T. B. (2000). Seed dispersal and movement patterns in two species of *Ceratogymna* hornbills in a West African tropical lowland forest. *Oecologia*, 125, 249-257.
97. Blake, S., Deem, S. L., Mossimbo, E., Maisels, F., & Walsh, P. (2009). Forest elephants: tree planters of the Congo. *Biotropica*, 41(4), 459-468.
98. Abedi-Lartey, M., Dechmann, D. K., Wikelski, M., Scharf, A. K., & Fahr, J. (2016). Long-distance seed dispersal by straw-coloured fruit bats varies by season and landscape. *Global Ecology and Conservation*, 7, 12-24.
99. Lehouck, V., Spanhove, T., Demeter, S., Groot, N. E., & Lens, L. (2009). Complementary seed dispersal by three avian frugivores in a fragmented Afromontane Forest. *Journal of Vegetation Science*, 20(6), 1110-1120.
100. Sato, H. (2018). Predictions of seed shadows generated by common brown lemurs (Eulemur fulvus) and their relationship to seasonal behavioral strategies*. International Journal of Primatology*, 39, 377-396.
101. Oleksy, R., Giuggioli, L., McKetterick, T. J., Racey, P. A., & Jones, G. (2017). Flying foxes create extensive seed shadows and enhance germination success of pioneer plant species in deforested Madagascan landscapes*. PLoS One*, 12(9), e0184023.
102. Lenz, J., Fiedler, W., Caprano, T., Friedrichs, W., Gaese, B. H., Wikelski, M., & Böhning-Gaese, K. (2011). Seed-dispersal distributions by trumpeter hornbills in fragmented landscapes. *Proceedings of the Royal Society B: Biological Sciences*, 278(1716), 2257-2264.
103. Mueller, T., Lenz, J., Caprano, T., Fiedler, W., & Böhning‐Gaese, K. (2014). Large frugivorous birds facilitate functional connectivity of fragmented landscapes*. Journal of applied ecology*, 51(3), 684-692.
104. Bunney, K., Bond, W. J., & Henley, M. (2017). Seed dispersal kernel of the largest surviving megaherbivore—the African savanna elephant. *Biotropica*, 49(3), 395-401.
105. Reynolds, C. (2016). The role of waterbirds in the dispersal of aquatic organisms in southern Africa.
106. Nakashima, Y., & Sukor, J. A. (2010). Importance of common palm civets *(Paradoxurus hermaphroditus)* as a long-distance disperser for large-seeded plants in degraded forests. *Tropics*, 18(4), 221-229.
107. Weir, J. E., & Corlett, R. T. (2007). How far do birds disperse seeds in the degraded tropical landscape of Hong Kong, China? *Landscape ecology*, 22, 131-140.
108. Naniwadekar, R., Rathore, A., Shukla, U., Chaplod, S., & Datta, A. (2019). How far do Asian forest hornbills disperse seeds? *Acta Oecologica*, 101, 103482.
109. Sekar, N., Lee, C. L., & Sukumar, R. (2015). In the elephant's seed shadow: the prospects of domestic bovids as replacement dispersers of three tropical Asian trees. *Ecology*, 96(8), 2093-2105.
110. Spiegel, O., & Nathan, R. (2007). Incorporating dispersal distance into the disperser effectiveness framework: frugivorous birds provide complementary dispersal to plants in a patchy environment. *Ecology letters*, 10(8), 718-728.
111. Tsoar, A., Shohami, D., & Nathan, R. (2010). A movement ecology approach to study seed dispersal and plant invasion: an overview and application of seed dispersal by fruit bats. *Fifty years of invasion ecology: the legacy of Charles Elton*, 101-119.
112. Nakamoto, A., Kinjo, K., & Izawa, M. (2009). The role of Orii’s flying-fox (*Pteropus dasymallus inopinatus*) as a pollinator and a seed disperser on Okinawa-jima Island, the Ryukyu Archipelago, Japan. *Ecological Research*, 24, 405-414.
113. Mise, Y., Yamazaki, K., Soga, M., & Koike, S. (2016). Comparing methods of acquiring mammalian endozoochorous seed dispersal distance distributions. *Ecological Research*, 31, 881-889.
114. Koike, S., Masaki, T., Nemoto, Y., Kozakai, C., Yamazaki, K., Kasai, S., & Kaji, K. (2011). Estimate of the seed shadow created by the Asiatic black bear *Ursus thibetanus* and its characteristics as a seed disperser in Japanese cool‐temperate forest. *Oikos*, 120(2), 280-290.
115. Soné, K., & Kohno, A. (1996). Application of radiotelemetry to the survey of acorn dispersal by *Apodemus* mice. *Ecological Research*, 11, 187-192.
116. Tamura, N., & Hayashi, F. (2008). Geographic variation in walnut seed size correlates with hoarding behaviour of two rodent species. *Ecological Research*, 23, 607-614.
117. Campos‐Arceiz, A., Traeholt, C., Jaffar, R., Santamaria, L., & Corlett, R. T. (2012). Asian tapirs are no elephants when it comes to seed dispersal. *Biotropica*, 44(2), 220-227.
118. Campos-Arceiz, A., Larrinaga, A. R., Weerasinghe, U. R., Takatsuki, S., Pastorini, J., Leimgruber, P., & Santamaría, L. (2008). Behavior rather than diet mediates seasonal differences in seed dispersal by Asian elephants. *Ecology*, 89(10), 2684-2691.
119. Murray, K. G. (1986). Avian seed dispersal of neotropical gap-dependent plants (Doctoral dissertation, University of Florida).
120. Westcott, D. A., & Graham, D. L. (2000). Patterns of movement and seed dispersal of a tropical frugivore. *Oecologia*, 122(2), 249-257.
121. Şekercioğlu, Ç. H., Loarie, S. R., Oviedo-Brenes, F., Mendenhall, C. D., Daily, G. C., & Ehrlich, P. R. (2015). Tropical countryside riparian corridors provide critical habitat and connectivity for seed-dispersing forest birds in a fragmented landscape*. Journal of Ornithology*, 156, 343-353.
122. Kays, R., Jansen, P. A., Knecht, E. M., Vohwinkel, R., & Wikelski, M. (2011). The effect of feeding time on dispersal of Virola seeds by toucans determined from GPS tracking and accelerometers. *Acta Oecologica*, 37(6), 625-631.
123. Hirsch, B. T., Kays, R., Pereira, V. E., & Jansen, P. A. (2012). Directed seed dispersal towards areas with low conspecific tree density by a scatter‐hoarding rodent. *Ecology Letters,* 15(12), 1423-1429.
124. Kempter, I., Nopp-Mayr, U., Hausleithner, C., & Gratzer, G. (2018). Tricky to track: comparing different tagging methods for tracing beechnut dispersal by small mammals. *Ecological Research*, 33, 1219-1231.
125. Pellerin, M., Picard, M., Saïd, S., Baubet, E., & Baltzinger, C. (2016). Complementary endozoochorous long-distance seed dispersal by three native herbivorous ungulates in Europe. *Basic and Applied Ecology*, 17(4), 321-332.
126. Breitbach, N., Böhning‐Gaese, K., Laube, I., & Schleuning, M. (2012). Short seed‐dispersal distances and low seedling recruitment in farmland populations of bird‐dispersed cherry trees. *Journal of Ecology*, 100(6), 1349-1358.
127. Kleyheeg, E. (2015). Seed dispersal by a generalist duck: ingestion, digestion and transportation by mallards (*Anas platyrhynchos*) (Doctoral dissertation, Utrecht University).
128. Kleyheeg, E., Fiedler, W., Safi, K., Waldenström, J., Wikelski, M., & Van Toor, M. L. (2019). A comprehensive model for the quantitative estimation of seed dispersal by migratory mallards*. Frontiers in Ecology and Evolution*, 7, 40.
129. Kurek, P., Dobrowolska, D., & Wiatrowska, B. (2019). Dispersal distance and burial mode of acorns in Eurasian Jays *Garrulus glandarius* in European temperate forests. *Acta Ornithologica*, 53(2), 155-162.
130. Piazzon, M., Larrinaga, A. R., Rodríguez‐Pérez, J., Latorre, L., Navarro, L., & Santamaría, L. (2012). Seed dispersal by lizards on a continental‐shelf island: predicting interspecific variation in seed rain based on plant distribution and lizard movement patterns. *Journal of Biogeography*, 39(11), 1984-1995.
131. Pons, J., & Pausas, J. G. (2007). Acorn dispersal estimated by radio-tracking. *Oecologia*, 153, 903-911.
132. Castro, J., Molina-Morales, M., Leverkus, A. B., Martínez-Baroja, L., Pérez-Camacho, L., Villar-Salvador, P., & Rey-Benayas, J. M. (2017). Effective nut dispersal by magpies (*Pica pica L.*) in a Mediterranean agroecosystem. *Oecologia*, 184, 183-192.
133. Morán-López, T., Alonso, C. L., & Díaz, M. (2015). Landscape effects on jay foraging behavior decrease acorn dispersal services in dehesas. *Acta Oecologica,* 69, 52-64.
134. Santamaria, L., Rodríguez-Pérez, J., Larrinaga, A. R., & Pias, B. (2007). Predicting spatial patterns of plant recruitment using animal-displacement kernels. *PLoS One*, 2(10), e1008.
135. Wu, J. X., Delparte, D. M., & Hart, P. J. (2014). Movement patterns of a native and non‐native frugivore in Hawaii and implications for seed dispersal. *Biotropica*, 46(2), 175-182.
136. Bartlow, A. W., Lichti, N. I., Curtis, R., Swihart, R. K., & Steele, M. A. (2018). Re-caching of acorns by rodents: Cache management in eastern deciduous forests of North America. *Acta Oecologica*, 92, 117-122.
137. Elza, M. C., Slover, C., & McGraw, J. B. (2016). Analysis of wood thrush (*Hylocichla mustelina*) movement patterns to explain the spatial structure of American ginseng (*Panax quinquefolius*) populations. *Ecological research*, 31(2), 195-201.
138. Rawsthorne, J., Watson, D. M., & Roshier, D. A. (2011). Implications of movement patterns of a dietary generalist for mistletoe seed dispersal. *Austral Ecology*, 36(6), 650-655.
139. Nield, A. P., Nathan, R., Enright, N. J., Ladd, P. G., & Perry, G. L. (2020). The spatial complexity of seed movement: Animal‐generated seed dispersal patterns in fragmented landscapes revealed by animal movement models. *Journal of Ecology*, 108(2), 687-701.
140. Ward, M. J., & Paton, D. C. (2007). Predicting mistletoe seed shadow and patterns of seed rain from movements of the mistletoebird, *Dicaeum hirundinaceum*. *Austral Ecology*, 32(2), 113-121.
141. Barea, L. P. (2008). Interactions between frugivores and their resources: case studies with the painted honeyeater *Grantiella picta* (Doctoral dissertation, Charles Sturt University).
142. Moore, G. J. (1991). Seed dispersal by male tooth-billed bowerbirds, *Scenopoeetes dentirostris (Ptilonorhynchidae*), in north-east Queensland rainforests: processes and consequences (Doctoral dissertation, James Cook University of North Queensland).
143. Westcott, D. A., Bentrupperbäumer, J., Bradford, M. G., & McKeown, A. (2005). Incorporating patterns of disperser behaviour into models of seed dispersal and its effects on estimated dispersal curves. *Oecologia*, 146, 57-67.
144. Thibault, M., Masse, F., Pujapujane, A., Lannuzel, G., Bordez, L., Potter, M. A., & Brescia, F. (2018). “Liaisons dangereuses”: The invasive red‐vented bulbul (*Pycnonotus cafer*), a disperser of exotic plant species in New Caledonia*. Ecology and evolution*, 8(18), 9259-9269.
145. Wotton, D. M., & Kelly, D. (2012). Do larger frugivores move seeds further? Body size, seed dispersal distance, and a case study of a large, sedentary pigeon. *Journal of Biogeography*, 39(11), 1973-1983.
146. Mack, A. L., & Druliner, G. (2003). A Non-Intrusive Method for Measuring Movements and Seed Dispersal in Cassowaries. *Journal of field ornithology*, 193-196.
147. Rehm, E., Fricke, E., Bender, J., Savidge, J., & Rogers, H. (2019). Animal movement drives variation in seed dispersal distance in a plant–animal network. *Proceedings of the Royal Society B*, 286(1894), 20182007.
148. Jerozolimski, A., Ribeiro, M. B. N., & Martins, M. (2009). Are tortoises important seed dispersers in Amazonian forests? *Oecologia*, 161, 517-528.
149. Uriarte, M., Anciães, M., Da Silva, M. T., Rubim, P., Johnson, E., & Bruna, E. M. (2011). Disentangling the drivers of reduced long‐distance seed dispersal by birds in an experimentally fragmented landscape. *Ecology*, 92(4), 924-937.
150. Franco, L. M., Fontúrbel, F. E., Guevara, G., & Soto-Gamboa, M. (2019). Movement behavior of the Monito del monte (*Dromiciops gliroides*): new insights into the ecology of a unique marsupial*. Revista chilena de historia natural*, 92.
151. Karubian, J., Duraes, R., Storey, J. L., & Smith, T. B. (2012). Mating Behavior Drives Seed Dispersal by the Long‐wattled Umbrellabird *Cephalopterus penduliger. Biotropica*, 44(5), 689-698.
152. Tori, W. P., Bodawatta, K. H., Tanager, K., Lewis, E. L., Neumeister, D. S., & Hogle, J. (2016). White-crowned manakin (*Dixiphia pipra*) use of space in the Ecuadorian Amazon. *Ornitol. Neotrop.*, 27, 145-154.
153. Holbrook, K. M. (2011). Home range and movement patterns of toucans: implications for seed dispersal. *Biotropica*, 43(3), 357-364.
154. Julien-Laferriere, D. (1993). Radio-tracking observations on ranging and foraging patterns by kinkajous *(Potos flavus)* in French Guiana. *Journal of Tropical Ecology*, 9(1), 19-32.
155. Guzmán, A., & Stevenson, P. (2008). Seed dispersal, habitat selection and movement patterns in the Amazonian tortoise, *Geochelone denticulata. Amphibia-Reptilia,* 29(4), 463-472.
156. Anderson, J. T., Nuttle, T., Saldaña Rojas, J. S., Pendergast, T. H., & Flecker, A. S. (2011). Extremely long-distance seed dispersal by an overfished Amazonian frugivore. *Proceedings of the Royal Society B: Biological Sciences*, 278(1723), 3329-3335.
157. Wróbel, A., Kurek, P., Bogdziewicz, M., Dobrowolska, D., & Zwolak, R. (2022). Avian dispersal of an invasive oak is modulated by acorn traits and the presence of a native oak. *Forest Ecology and Management*, 505, 119866.
158. Poulsen, J. R., Beirne, C., Rundel, C., Baldino, M., Kim, S., Knorr, J., ... & Wright, J. P. (2021). Long distance seed dispersal by forest elephants. *Frontiers in Ecology and Evolution*, 9, 789264.
159. Stevenson, P. R., Cardona, L., Cárdenas, S., & Link, A. (2021). Oilbirds disperse large seeds at longer distance than extinct megafauna. *Scientific Reports*, 11(1), 420.
160. Martín-Vélez, V., Montalvo, T., Afán, I., Sánchez-Márquez, A., Aymí, R., Figuerola, J., & Navarro, J. (2022). Gulls living in cities as overlooked seed dispersers within and outside urban environments. *Science of the Total Environment*, 823, 153535.
161. Karraker, N. E., Dikari Kusrini, M., Atutubo, J. R., Healey, R. M., & Yusratul, A. (2020). Non‐marine turtle plays important functional roles in Indonesian ecosystems. *Ecology and Evolution*, 10(18), 9613-9623.
162. Martínez‐Baroja, L., Pérez‐Camacho, L., Villar‐Salvador, P., Rebollo, S., Leverkus, A. B., Pesendorfer, M. B., & Rey‐Benayas, J. M. (2021). Caching territoriality and site preferences by a scatter‐hoarder drive the spatial pattern of seed dispersal and affect seedling emergence. *Journal of Ecology*, 109(6), 2342-2353.
